# Avian haemosporidian parasites of accipitriform raptors

**DOI:** 10.1186/s12936-021-04019-z

**Published:** 2022-01-05

**Authors:** Josef Harl, Tanja Himmel, Gediminas Valkiūnas, Mikas Ilgūnas, Nora Nedorost, Julia Matt, Anna Kübber-Heiss, Amer Alic, Cornelia Konicek, Herbert Weissenböck

**Affiliations:** 1 Institute of Pathology, Department of Pathobiology, University of Veterinary Medicine Vienna, Veterinaerplatz 1, 1210 Vienna, Austria; 2 Nature Research Centre, Akademijos 2, 08412 Vilnius, Lithuania; 3 Research Institute of Wildlife Ecology, Department of Integrative Biology and Evolution, University of Veterinary Medicine Vienna, Savoyenstraße 1, 1160 Vienna, Austria; 4 Department of Pathology, Faculty of Veterinary Medicine, University of Sarajevo, Zmaja od Bosne 90, 71000 Sarajevo, Bosnia and Herzegovina; 5 Service for Birds and Reptiles, Clinic for Small Animal Internal Medicine, Department for Companion Animals and Horses, University of Veterinary Medicine Vienna, Veterinaerplatz 1, 1210 Vienna, Austria

## Abstract

**Background:**

The order Accipitriformes comprises the largest group of birds of prey with 260 species in four families. So far, 21 haemosporidian parasite species have been described from or reported to occur in accipitriform birds. Only five of these parasite species have been characterized molecular genetically. The first part of this study involved molecular genetic screening of accipitriform raptors from Austria and Bosnia-Herzegovina and the first chromogenic in situ hybridization approach targeting parasites in this host group. The aim of the second part of this study was to summarize the *CytB* sequence data of haemosporidian parasites from accipitriform raptors and to visualize the geographic and host distribution of the lineages.

**Methods:**

Blood and tissue samples of 183 accipitriform raptors from Austria and Bosnia-Herzegovina were screened for *Plasmodium*, *Haemoproteus* and *Leucocytozoon* parasites by nested PCR, and tissue samples of 23 PCR-positive birds were subjected to chromogenic in situ hybridization using genus-specific probes targeting the parasites’ *18S* rRNAs. All published *CytB* sequence data from accipitriform raptors were analysed, phylogenetic trees were calculated, and DNA haplotype network analyses were performed with sequences from clades featuring multiple lineages detected in this host group.

**Results:**

Of the 183 raptors from Austria and Bosnia-Herzegovina screened by PCR and sequencing, 80 individuals (44%) were infected with haemosporidian parasites. Among the 39 *CytB* lineages detected, 18 were found for the first time in the present study. The chromogenic in situ hybridization revealed exo-erythrocytic tissue stages of *Leucocytozoon* parasites belonging to the *Leucocytozoon toddi* species group in the kidneys of 14 infected birds. The total number of *CytB* lineages recorded in accipitriform birds worldwide was 57 for *Leucocytozoon*, 25 for *Plasmodium*, and 21 for *Haemoproteus*.

**Conclusion:**

The analysis of the DNA haplotype networks allowed identifying numerous distinct groups of lineages, which have not yet been linked to morphospecies, and many of them likely belong to yet undescribed parasite species. Tissue stages of *Leucocytozoon* parasites developing in accipitriform raptors were discovered and described. The majority of *Leucocytozoon* and *Haemoproteus* lineages are specific to this host group, but most *Plasmodium* lineages were found in birds of other orders. This might indicate local transmission from birds kept at the same facilities (raptor rescue centres and zoos), likely resulting in abortive infections. To clarify the taxonomic and systematic problems, combined morphological and molecular genetic analyses on a wider range of accipitriform host species are needed.

**Supplementary Information:**

The online version contains supplementary material available at 10.1186/s12936-021-04019-z.

## Background

The order Haemosporida (phylum Apicomplexa) includes several genera of single-celled eukaryotic parasites, which infect vertebrate hosts and are transmitted by blood-sucking dipteran vectors. Haemosporidian parasites feature complex life cycles and undergo multiple developmental stages in both the vertebrate hosts and dipteran vectors. The infection of tissue and blood cells can seriously affect the host’s health, potentially leading to the damage of organs [1, 2], anaemia, dyspnoea, and death [3]. Birds feature the largest diversity of haemosporidian parasites with more than 250 species classified into the genera *Plasmodium*, *Haemoproteus*, *Leucocytozoon*, and *Fallisia* [4]. Traditionally, haemosporidian parasite species were characterized based on the morphology of blood stages, an approach, which is limited by the low number of morphological features, particularly in species of *Leucocytozoon* and *Akiba*. The introduction of molecular genetics opened new possibilities to characterize the diversity of avian haemosporidians. DNA barcoding assays were developed two decades ago and a 478 base pair (bp) section of the mitochondrial *Cytochrome B* (*CytB*) was established as the main reference sequence for the identification of haemosporidian lineages [5, 6]. Bensch et al*.* [7] developed the MalAvi database (http://130.235.244.92/Malavi/), which uses a standardized nomenclature to assign unique names to *CytB* lineages of avian haemosporidians and summarizes data on hosts, localities and references of most recorded lineages, providing an important source of information for the haemosporidian research community. The MalAvi database currently features a collection of about 4,000 unique *CytB* lineages recorded in over 35,000 birds. Only 279 records originate from accipitriform raptors (Accipitriformes), two-thirds of which were published in the following eight publications: Pérez-Rodríguez et al*.* [8], Sehgal et al*.* [9], Jasper et al*.* [10], Huang et al*.* [11], Hanel et al*.* [12], Poharkar et al*.* [13], Krone et al*.* [14], and Ciloglu et al*.* [15]. Avian raptors are under-represented in haemosporidian studies because they have low population densities and are usually not caught in mist nets like many passeriform birds. Moreover, many raptor species are protected by conservation laws and, therefore, are rarely, if at all, used in experimental studies. As a result, many aspects of haemosporidioses in accipitriform raptors are poorly studied, particularly the tissue stages, which develop in the organs.

The bird order Accipitriformes represents the largest group of birds of prey with 260 species in four families, compared to Strigiformes with 234 species in two families, and Falconiformes with 66 species in one family. Within the order Accipitriformes, the family Accipitridae is most diverse with 250 species in 69 genera including hawks, eagles, kites and others; the family Cathartidae includes nine species of New World vultures in six genera; the monotypic family Sagittariidae features only the secretary bird *Sagittarius serpentarius*; the osprey *Pandion haliaetus* is the only two species of the family Pandionidae (http://datazone.birdlife.org/).

The number of haemosporidian parasites described from accipitriform hosts is low with 10 *Leucocytozoon*, five *Haemoproteus*, and three *Plasmodium* species (Table 1). *Plasmodium circumflexum* (type host: *Turdus pilaris*), *Plasmodium fallax* (type host: *Strix woodfordii*), and *Plasmodium forresteri* (type host: *Strix woodfordii*) are included in the table because morphologically indistinguishable parasites were reported to be common in accipitriform raptors [3]. *Plasmodium alloelongatum* and *Plasmodium buteonis* are listed in the table but the former name is likely a synonym of *Plasmodium elongatum.* Blood stages of *P. buteonis* should be re-examined because this name was considered a species inquirenda in the latest taxonomic review [16]. All 10 *Leucocytozoon* species belong to the *Leucocytozoon toddi* group, whose species exclusively infect accipitriform raptors. However, most of these species were synonymized with *L. toddi* because their blood stages and host cells possess similar characters and could not always be reliably delimitated by their morphological features [17–20]. The status of the current synonyms may be changed when more information on the parasites’ life cycles and DNA sequences are available. Studies analysing both mitochondrial and nuclear genes of *Haemoproteus* spp. and *Leucocytozoon* spp. found that closely related lineages, differing in one or a few bp in the *CytB*, were strictly associated with unique alleles for the nuclear loci, thus supporting the presence of a high number of cryptic species [9, 21, 22].

**Table 1:** Haemosporidian parasite species described from or reported commonly in accipitriform birds

Parasite species	Authors	Type host (bold) and additional hosts	Type locality
*Haemoproteus buteonis*	Wingstrand, 1947	***Buteo buteo***, (*Accipiter cooperii, Accipiter nisus, Aquila nipalensis Buteo platypterus, Buteo rufinus, Circus aeruginosus, Pernis apivorus, Pernis ptilorhynchus*)	Sweden
*Haemoproteus catharti* hCATAUR01	Greiner et al. 2011	***Cathartes aura***	South Carolina (USA)
*Haemoproteus elani* hBUBT1	Mello, 1935	***Elanus caeruleus***, (*Accipiter cooperii, Accipiter gentilis, Accipiter melanoleucus, Accipiter nisus, Accipiter striatus, Aegypius tracheliotus, Aquila rapax, Buteo jamaicensis, Buteo lagopus, Buteo lineatus, Circaetus gallicus, Gyps africanus, Hieraaetus fasciatus*)	Goa (western India)
*Haemoproteus janovyi*	Greiner and Mundy, 1979	***Gyps africanus***, (*Melierax canorus, Necrosyrtes monachus, Torgos tracheliotus, Trigonoceps occipitalis*)	Northwest Zimbabwe
*Haemoproteus nisi*	Peirce and Marquiss, 1983	***Accipiter nisus****,* (*Accipiter cooperi, Accipiter soloensis, Accipiter striatus, Accipiter tachiro, Accipiter trivirgatus, Accipiter virgatus, Aquila clanga, Aquila wahlbergi, Butastur indicus, Buteo buteo, Buteo jamaicensis, Circus aeruginosus, Circus cyaneus, Circus macrourus, Circus pygargus, Melierax metabates, Milvus migrans*)	Scotland (UK)
*Leucocytozoon audieri*	Laveran and Nattan-Larrier, 1911	***Haliaeetus vocifer***	Congo
*Leucocytozoon bacelari*	Tendeiro, 1947	***Kaupifalco monogrammicus***	Guinea Bissau
*Leucocytozoon beaurepairei*	Travassos Santos Diaz, 1954	***Sagittarius serpentarius***	Mozambique
*Leucocytozoon buteonis* lBUBT2, lBUTJAM10, lBUTREG01	Coatney and Roudabush, 1937	***Buteo jamaicensis***, (*Buteo buteo, Buteo regalis, Buteo lineatus*)	Nebraska (USA)
*Leucocytozoon circaeti*	Sergent and Fabiani, 1922	***Circaetus gallicus***	Algeria
*Leucocytozoon franchini*	França, 1927	***Circus macrourus*** (‘albanella pallida’)	Italy
*Leucocytozoon martyi*	Commes, 1918	***Accipiter badius sphenurus***	Mali
*Leucocytozoon mathisi* lACCOP01, lACNI04	França, 1912	***Accipiter nisus***, (*Accipiter cooperii*)	Portugal
*Leucocytozoon muratovi*	Subkhonov, 1980	***Circus aeruginosus***	Tajikistan
*Leucocytozoon toddi*	Sambon, 1908	***Kaupifalco monogrammicus***, (numerous birds of the Accipitriformes)	Congo
*Plasmodium circumflexum* pTURDUS1, pBT7	Kikuth, 1931	***Turdus pilaris***, (Passeriformes, Accipitriformes, Anseriformes, Columbiformes, Coraciiformes, Charadriiformes, Falconiformes, Strigiformes, Galliformes, and some others)	Germany
*Plasmodium fallax*	Schwetz, 1930	***Strix woodfordii***, (*Accipiter nisus, Aquila rapax, Aquila wahlbergi, Gyps africanus*)	Belgian Congo
*Plasmodium forresteri*	Telford, Nayar, Foster & Knight, 1997	***Strix varia***, (*Buteo jamaicensis, Buteo lineatus, Buteo platypterus, Haliaeetus leucocephalus*)	Georgia (USA)
*Plasmodium accipiteris*	Paperna, Yosef & Landau	***Accipiter brevipes***	Eilat (Israel)
Plasmodium alloelongatum*	Paperna, Yosef & Landau	***Accipiter brevipes***	Eilat (Israel)
Plasmodium buteonis*	Paperna, Yosef & Landau	***Buteo buteo***	Eilat (Israel)

Haemosporidian parasite species described from or reported commonly in accipitriform birds based on morphological blood stage records. If *CytB* lineages were linked to morphospecies, the MalAvi lineage name is indicated following the species name. The names of type hosts are written in bold letters, additional hosts are reported in brackets

*These two names are likely invalid according to [16]

The present study consists of two parts. The first represents a molecular screening approach for which 183 accipitriform raptors of 16 species from Austria and Bosnia-Herzegovina (B.-H.) were screened for avian haemosporidians by PCR and sequencing the standard *CytB* barcode region. The present study not only included the most diverse sample of accipitriform raptors in Europe, but it also represents the first molecular genetic approach targeting haemosporidian parasites in the organs of infected raptors by chromogenic in situ hybridization (CISH). This study aimed at investigating the diversity of haemosporidian lineages in accipitriform raptors from Central Europe, examining blood and tissue stages in the hosts’ organs by CISH, and identifying potential pathological effects of exo-erythrocytic development (tissue merogony) in this host group.

The second part of the study aims at analysing the geographic and host distribution of haemosporidian parasite lineages in accipitriform raptors worldwide. It follows the approach of [4], who summarized information on avian haemosporidian parasites of the family Turdidae. Other records were gathered from NCBI GenBank, MalAvi database and related publications. Phylogenetic clades containing multiple *CytB* lineages detected in accipitriform raptors were identified and DNA haplotype networks were calculated to visualize their geographic and host distribution. Moreover, information on morphologically but not yet molecular genetically characterized parasite species is discussed. This approach was selected to estimate the potential number of haemosporidian parasite species in this host group, identify lineages, which potentially belong to yet unrecognized parasite species, and create a basis for comparing *CytB* data with taxonomic information pre-dating the molecular genetic era.

## Methods

### Molecular screening of accipitriform raptors from Austria and Bosnia-Herzegovina

#### Sample preparation

For the present study, blood and tissue samples from 183 individual accipitriform raptors of 16 species were collected in Austria and B.-H. The samples collected by different institutions originated from the following host species: *Accipiter gentilis* (11 individuals), *Accipiter nisus* (25), *Aquila chrysaetos* (1), *Aquila heliaca* (10), *Buteo buteo* (75), *Buteo lagopus* (1), *Buteo* sp. (5), *Circus aeruginosus* (22), *Circus cyaneus* (4), *Clanga pomarina* (2), *Gypaetus barbatus* (2), *Gyps fulvus* (2), *Haliaeetus albicilla* (12), *Haliaeetus leucocephalus* (1), *Milvus milvus* (8), *Pandion haliaetus* (1), and *Pernis apivorus* (1).

The Research Institute of Wildlife Ecology (Department of Interdisciplinary Life Sciences, Vetmeduni Vienna) provided frozen and formalin-fixed paraffin-embedded (FFPE) tissue samples of 112 birds collected between 2009 and 2018 in Austria (mostly Lower Austria, Upper Austria and Burgenland). Paraffin blocks of individual birds contained tissues of the heart, lung, liver, and spleen and in most cases also brain, spleen, skeletal muscle, and intestines. Blood samples were taken from 58 living birds received for treatment at the service unit for birds and reptiles of the clinic for small animal internal medicine (Department for Companion Animals and Horses, Vetmeduni Vienna) between 2015 and 2016. The clinical records showed that more than half of the birds suffered from traumatic injuries (e.g., bird strikes) and neurological problems. Blood counts, tests for other pathogens, and thorough examinations were performed only in a few cases, therefore, this information is not provided here. The blood was taken by puncturing the brachial vein using heparinized microcapillaries to transfer blood drops to high-grade filter papers Whatman™ 903 (GE Healthcare, Buckinghamshire, UK). Frozen tissue samples (liver and lung) of 11 birds were collected by the Department of Pathology at the Sarajevo Faculty of Veterinary Medicine (University of Sarajevo, B.-H.) between 2017 and 2018. Twelve birds, which died after treatment at the service unit for birds and reptiles (Vetmeduni Vienna), were dissected at the Institute of Pathology (Department of Pathobiology, Vetmeduni Vienna) and tissue samples were taken from the heart, lung, liver, spleen, kidney, brain, skeletal muscle, and gastrointestinal tract. For histology, tissue samples were fixed in formalin and embedded in paraffin. For molecular analyses, liver, spleen and brain samples were frozen and stored at − 80 °C until further use. All FFPE tissue blocks, frozen tissue samples, blood films, and blood spots are stored in the pathological collections of either the Research Institute of Wildlife Ecology (Vetmeduni Vienna), the Institute of Pathology (Vetmeduni Vienna), or the Department of Pathology at the Sarajevo Faculty of Veterinary Medicine.

#### DNA extraction, PCRs, and sequence analyses

The DNA of all samples was extracted either from tissue (liver and spleen) or blood spots using the DNeasy Blood & Tissue Kit (QIAGEN, Venlo, The Netherlands) by following the manufacturer’s protocol for isolation of DNA from tissue samples. Two 100 µl eluates were made from the same column in the last centrifugation step, the first at 8000 rpm and the second at 13,000 rpm. The DNA samples (second eluate) of all 183 individuals were screened for the presence of avian haemosporidians using the nested PCR protocol established by [6], which allows the amplification of 478/476 bp sections of the mitochondrial *CytB* gene in *Plasmodium*, *Haemoproteus* and *Leucocytozoon* parasites. The primers HaemNFI (5′-CAT ATA TTA AGA GAA NTA TGG AG-3′) and HaemNR3 (5′-ATA GAA AGAT AAG AAA TAC CAT TC-3′) were used in the first PCR. In the nested PCRs, the primers HaemF (5′-ATG GTG CTT TCG ATA TAT GCA TG-3′) and HaemR2 (5′-GCA TTA TCT GGA TGT GAT AAT GGT-3′) were used to amplify a 478 bp section in *Plasmodium* spp. and *Haemoproteus* spp., and HaemFL (5′-ATG GTG TTT TAG ATA CTT ACA TT-3′) and HaemR2L (5′-CAT TAT CTG GAT GAG ATA ATG GIG C-3′) were used to amplify a 476 bp section in *Leucocytozoon* spp. The nested PCR assay by [6] does not allow amplification of the *CytB* in parasites of the *L. toddi* group. In particular, the forward primer of the first PCR, HaemNFI, shows a two bp mismatch at the 3’-end compared to *L. toddi* group sequences (AG *vs* GC), and the nested reverse primer HaemR2L, used for amplification of the *CytB* in *Leucocytozoon* parasites, features a two bp mismatch at the 3’-end (GC [complement] *vs* CT, TT, or AT). Therefore, all samples were screened also using the nested PCR protocol established by [23], which allows the amplification of a 528 bp fragment specifically of parasites in this group. The primers CytB_L2_F (5′-GAG AGT TAT GGG CTG GAT GGT-3′) and CytB_L2_R (5′-TAG AAA GCC AAG AAA TAC CAT TCT G-3′) were used in the first PCR, and the primers CytB_L2_nF (5′-GCT GGA TGG TGT TTT AGA TAY ATG C-3′) and CytB_L2_nR (5′-CCA TTC TGG AAC AAT ATG TAA AGG TG-3′) were used in the nested PCR.

All PCRs were performed using the GoTaq® G2 Flexi DNA Polymerase (Promega, Madison, WI, USA). They were conducted in 25 µl volumes containing 14.375 µl nuclease-free water, 5 µl 5X Green GoTaq Flexi Buffer, 2 µl MgCl_2_ solution (25 mM), 0.5 µl nucleotide mix (10 mM), 0.125 µl GoTaq G2 Flexi DNA Polymerase (5 µ/µl), each 1 µl forward and reverse primer (10 mM), and 1 µl of DNA template. The PCRs started with an initial denaturation for 2 min at 94 °C, followed by 35 cycles with 30 s at 94 °C, 30 s at the respective annealing temperatures (50 °C: HaemNFI/ HaemNR3, HaemF/HaemR2, and HaemFL/HaemR2L; 55 °C: CytB_L2_F/ CytB_L2_F and CytB_L2_nF/ CytB_L2_nR), 1 min at 72 °C, and a final extension for 10 min at 72 °C. Each 1 µl of the first PCR product was used as a template in the nested PCRs. Negative and positive controls (previously confirmed by sequencing) were included in all PCRs. The PCR products were visualized on 1% agarose gels stained with MIDORI^Green^ Advance DNA/RNA stain (Nippon Genetics Europe, Düren, Germany). All positive products from the nested PCRs were sent to Microsynth Austria (Vienna, Austria) for purification and sequencing in both directions using the respective PCR primers. The forward and reverse sequences were aligned with Bioedit v. 7.0.5.3 [24], and the electropherograms were carefully checked for double peaks to identify mixed infections. The positions featuring double peaks in both the forward and reverse sequences were coded with the corresponding ambiguity codes. Then these sequences were unphased both manually and with DnaSP v.6.12.3 [25] based on an alignment containing all sequences generated for the present study. To confirm their identity and compare their similarity with already published data, the sequences were subjected to BLAST searches on NCBI GenBank and the avian malaria database MalAvi [7]. *CytB* sequences of new lineages and information on hosts and geographic origin were added to the MalAvi database, and all sequences were also uploaded onto NCBI GenBank (accession numbers OL598427–OL598534).

#### Chromogenic in situ hybridization

Chromogenic in situ hybridization (CISH) was performed on tissue samples of 33 PCR-positive birds of which paraffin blocks were available. For detecting haemosporidian parasite stages in tissue sections of these birds, 1–2 µm histological sections were prepared, one of which was stained with haematoxylin and eosin (HE), and the remaining were subjected to CISH. CISH was performed by following previously established protocols and using (sub)genus-specific probes, which target the *18S* ribosomal RNAs of parasites from the genera *Plasmodium*, *Haemoproteus*, and *Leucocytozoon* [2, 23]. Specifically, the following probes were used: Plas18S (5′-TTT AAT AAC TCG TTA TAT ATA TCA GTG TAG CAC-3′) for detecting *Plasmodium* spp., Haemo18S_1 (5′-GCT AAC CGT AGT TAT AGT CGC CAT CTC-3′) for *Haemoproteus* parasites of the subgenus *Parahaemoproteus*, Leuco18S_1 (5′-TAG GAC TCC CCA CTT GTC TTT TTC TTGA-3′) for *Leucocytozoon* parasites of the subgenus *Leucocytozoon*, and Ltod18S (5′-GCT AAC CGT AGT TAT AGT CGC CAT CTC-3′) for targeting parasites of the *L. toddi* species group. In cases of mixed infections, several tissue sections were separately incubated with all relevant probes. All HE-stained sections and in situ hybridized sections were examined at 50x-1000 × magnification using an Olympus BX51 microscope (Olympus Europa, Hamburg, Germany) equipped with an Olympus DP71 camera for microphotography. Images were adjusted for brightness and contrast and assembled in Adobe Photoshop CC 2021 (Adobe, San José, CA, USA).

### Diversity of haemosporidian *CytB* lineages in accipitriform raptors worldwide

#### Collection of *CytB* data from GenBank and MalAvi databases

The second part of the present study provides a summary of data on avian haemosporidian parasite lineages of accipitriform raptors worldwide. For a previous study on haemosporidian parasites in birds of the family Turdidae [4], the first author (JH) collected all haemosporidian *CytB* sequences and related information available on NCBI GenBank. This dataset was used to identify clades featuring similar lineages by sorting the sequences with MAFFT v.7 [26], manually inspecting the alignments using Bioedit v.7.0.8.0 [24], and performing Maximum Likelihood (ML) analyses on the W-IQ-TREE web server (http://iqtree.cibiv.univie.ac.at/; [27]), as described in [4]. New data published to February 2021 were added to this data set. To incorporate information from the MalAvi database (http://130.235.244.92/Malavi/; [7]), the ‘host and sites table’ was mined for all data originating from accipitriform hosts (currently classified wrongly as Falconiformes in the MalAvi database). This information was then added to a Microsoft Excel spreadsheet containing the GenBank data and the new sequence data generated for the present study. Based on the combined data from NCBI GenBank and the MalAvi database, the authors evaluated all sequence data originating from accipitriform hosts, identified those clades featuring multiple haemosporidian parasite lineages detected in accipitriform hosts, and extracted information on all lineages contained within these clades (including data on non-accipitriform birds) from the MalAvi database.

#### Phylogenetic analysis of genus clades and *Leucocytozoon toddi* group

A phylogenetic tree was calculated with lineages belonging to the *L. toddi* species group, the most diverse group of haemosporidian parasites in accipitriform raptors. The alignment contained all *L. toddi* group lineages covering the full 478 bp *CytB* fragment of the DNA barcode region, 48 lineages in total (including 16 new ones detected in the present study). To conform to the alignments used for the DNA haplotype networks, the first and last two bp of the alignment were trimmed prior to the phylogenetic analyses. This was done with all alignments analysed for the present study because the end parts of many published sequences were not curated thoroughly and contained some obvious errors. The sequence of *Leucocytozoon californicus* lCIAE02 (accession number EF607287) was used as an outgroup. A ML bootstrap consensus tree (1,000 replicates) was calculated using the W-IQ-TREE web server (http://iqtree.cibiv.univie.ac.at/; [27]), applying the model TIM2 + G4, which was suggested as best fit for the data set in the model test according to the Bayesian inference criterion (BIC). A Bayesian Inference (BI) tree was calculated with MrBayes v.3.2.2 [28]. Applying the model GTR + G, the BI analysis was run for 5^10^ generations (2 runs with 4 chains, one of which was heated), sampling every thousandth tree. The first 25% of the trees were discarded as burn-in and a majority rule consensus tree was calculated from the remaining 3,750 trees. The tree was visualized with Figtree v.1.4.4 (http://tree.bio.ed.ac.uk/software/figtree/; Andrew Rambaut) and finalized with Adobe Illustrator CC v.2015 (Adobe Inc., San José, CA, USA). Phylogenetic trees were also calculated for the *Plasmodium*, *Haemoproteus* and other *Leucocytozoon* lineages, including related lineages from other bird hosts contained in the DNA haplotype networks. BI and ML trees were calculated using the same settings as for the sequences of the *L. toddi* species group but with varying substitution models. The models used for the ML analyses were TIM2 + G4 + I (BI: GTR + G + I) for *Plasmodium* and *Haemoproteus*, and TIM2 + G4 (BI: GTR + G) for *Leucocytozoon*.

#### DNA haplotype networks

DNA haplotype networks were calculated for clades featuring multiple lineages of haemosporidian parasites from accipitriform birds. For each *CytB* lineage contained in the networks, information on the number of hosts and their geographic origin were obtained from NCBI GenBank, the MalAvi ‘host and sites table’, and the respective publications. All GenBank sequences, which contained ambiguous characters, obvious sequencing errors, or which did not cover the 474 bp *CytB* section used in the analysis were removed from the alignments. There were several cases in which lineage names and related information were reported only to the MalAvi database, but sequences were not submitted to NCBI GenBank. Although a quality check could not be performed for these data, they were still included to obtain a more comprehensive picture. The sequences of a few lineages, which were deposited only in NCBI GenBank but not published in research studies, were also included because they featured valuable information. Analyses were performed with the sequence data of nine *Leucocytozoon* clades, eight of which belong to the *L. toddi* species group, three *Plasmodium* clades, and four *Haemoproteus* clades. The DNA haplotype network analyses were performed following the procedure of [4]: (1) The alignments were trimmed to 474 bp by removing the first and last two base pairs of the full 478 bp barcode section because particularly the end parts of many published sequences were not curated thoroughly and contained some obvious errors; (2) Median-Joining haplotype networks were calculated with Network 10.2.0.0 (Fluxus Technology Ltd, Suffolk, UK) applying the default settings; (3) Using Network Publisher v.2.1.2.5 (Fluxus Technology Ltd), the networks were graphically arranged and information on the host species and geographic regions was added. For each network, two visual representations were prepared, the first showing the host distribution of the lineages, the second showing the geographic distribution according to the United Nations Geoscheme; and, (4) The networks were finalized with Adobe Illustrator CC v.2015 (Adobe Inc., San José, CA, USA).

## Results

### Molecular study on accipitriform raptors from Austria and Bosnia-Herzegovina

#### Diversity and infection rates of haemosporidian parasite lineages

For this part of the study, samples of 183 accipitriform raptors of 16 species were screened for the presence of avian haemosporidians. In total, 80 individuals (44%) featured haemosporidian infections, of which 49 were mono-infections, 27 double infections, and four triple infections (Table 2). Infections with parasites of the *L. toddi* species group were most common with 60 birds either being infected with one (43 individuals) or two lineages (16). The most common lineage was lBUBT2 (27), followed by lBUTBUT03 (13), lBUTBUT07 (6), lCIAE03 (4), lMILVUS01 (4), lACNI04 (3), lMILANS04 (2), lBUTBUT08 (2), lACCGEN01 (1), and lACNI1 (1). Apart from the novel lineages lBUTBUT07 and lBUTBUT08, nine additional new *L. toddi* group lineages were found in one bird each: lCLAPOM02, lCLAPOM03, lBUTBUT13, lCIAE04, lCIAE05, lBUTBUT11, lBUTBUT12, lBUTBUT09, and lBUTBUT10. *Leucocytozoon* sp. lCIAE02 was detected in four individuals of *Circus aeruginosus*, whereby one individual featured a double infection with *Leucocytozoon* sp. lCIAE06. *Leucocytozoon* sp. lMILVUS2 was found in each one individual of *Buteo buteo* and *Buteo lagopus*, and *Leucocytozoon* sp. lASOT06 in each one individual of *Buteo buteo* and *Circus aeruginosus*. *Plasmodium circumflexum* was by far the most common *Plasmodium* parasite with 11 individuals being infected with the lineage pTURDUS1, two with pBT7, and one individual was infected with both pTURDUS1 and the new lineage pCIAE07, differing in one bp from pTURDUS1. One bird each was infected with the *Plasmodium* lineages *P. elongatum* pGRW06 (*Buteo buteo*), *P.* cf. *elongatum* pMILANS05 (*Circus aeruginosus*), *Plasmodium matutinum* pLINN1 (*Accipiter gentilis*), and *Plasmodium* sp. pSYBOR10 (*Circus aeruginosus*). One bird each featured *Haemoproteus* infections with *Haemoproteus brachiatus* lLK03 (*Circus aeruginosus*), *Haemoproteus noctuae* hCIRCUM01 (*Circus aeruginosus*), and *Haemoproteus* aff. *elani* hCIAE08 (*Circus aeruginosus*), and two individuals of *Buteo buteo* featured *Haemoproteus elani* hBUBT1 (*Buteo buteo*).

**Table 2:** Haemosporidian *CytB* lineages found in accipitriform raptors from Austria and Bosnia-Herzegovina

Host species	n tested	n infected	*Plasmodium*	*Haemoproteus*	*Leucocytozoon*
*Accipiter gentilis*	11	5	*P. matutinum* pLINN1 (1)	–	*L. buteonis* lBUBT2 (1), *L.* sp. lBUTBUT03 (2), *L*. sp. lACCGEN01 (1)
*Accipiter nisus*	25	12	*P. circumflexum* pTURDUS1 (3)	–	*L*. sp. lACNI03 (1), *L*. sp. **lACNI05** (1), *L*. *mathisi* lACNI04 (2), *L*. sp. lACNI1 (1), *L*. sp. **lACNI06** (1), *L*. sp. **lACNI07** (1), *L*. sp. lMILANS04 (2)
*Aquila chrysaetos*	1	–	–	–	–
*Aquila heliaca*	10	–	–	–	–
*Buteo buteo*	75	47	*P. circumflexum* pTURDUS1 (2), *P. circumflexum* pBT7 (2), *P. elongatum* pGRW06 (1)	*H. noctuae* hCIRCUM01 (1), *H. elani* hBUBT1 (2)	*L*. sp. lASOT06 (1), *L*. *buteonis* lBUBT2 (25), *L*. sp. **lBUTBUT07** (5), *L*. sp. **lBUTBUT08** (2), *L*. sp. **lBUTBUT09** (1), *L*. sp. **lBUTBUT10** (1), *L*. sp. lBUTBUT03 (12), *L*. sp. **lBUTBUT11** (1), *L*. sp. **lBUTBUT12** (1), *L*. sp. lMILVUS01 (4), *L*. sp. **lBUTBUT13** (1), *L*. sp. lMILVUS2 (1)
*Buteo lagopus*	1	1	–	–	*L*. sp. lMILVUS02 (1), *L*. sp. **lBUTBUT07** (1)
*Buteo* sp.	5	–	–	–	–
*Circus aeruginosus*	22	15	*P. circumflexum* pTURDUS1 (5), *P*. cf. *circumflexum* **pCIAE07** (1), *P*. sp. pSYBOR10 (1)	*H. brachiatus* hLK03 (1), *H.* aff. *elani* **hCIAE08** (1)	*L*. *mathisi* lACNI04 (1), *L*. sp. lASOT06 (1), *L*. *buteonis* lBUBT2 (1), *L*. sp. **lCIAE05** (1), *L*. sp. lCIAE02 (5), *L*. sp. **lCIAE06** (1), *L*. sp. lCIAE03 (5), *L*. sp. **lCIAE04** (1)
*Circus cyaneus*	4	1	–	–	*L*. sp. **lCIRCYA01** (1)
*Clanga pomarina*	2	1	–	–	*L*. sp. **lCLAPOM02** (1), *L*. sp. **lCLAPOM03** (1)
*Gypaetus barbatus*	2	-	–	–	–
*Gyps fulvus*	2	-	–	–	–
*Haliaeetus albicilla*	12	1	–	–	*L*. sp. lMILVUS02 (1)
*H. leucocephalus*	1		–	–	–
*Milvus milvus*	8		–	–	–
*Pernis apivorus*	1		–	–	–
*Pandion haliaetus*	1		–	–	–

Names of parasite species are indicated if lineages were already linked to morphospecies. The numbers in the brackets indicate the number of individuals featuring the respective lineages. Lineages detected for the first time in the present study are highlighted in bold letters

The term ‘aff.’ (‘species affinis’) indicates that the lineage is similar to other lineages, which were already linked to morphospecies

#### Parasites stages detected by CISH

**Fig. 1:**
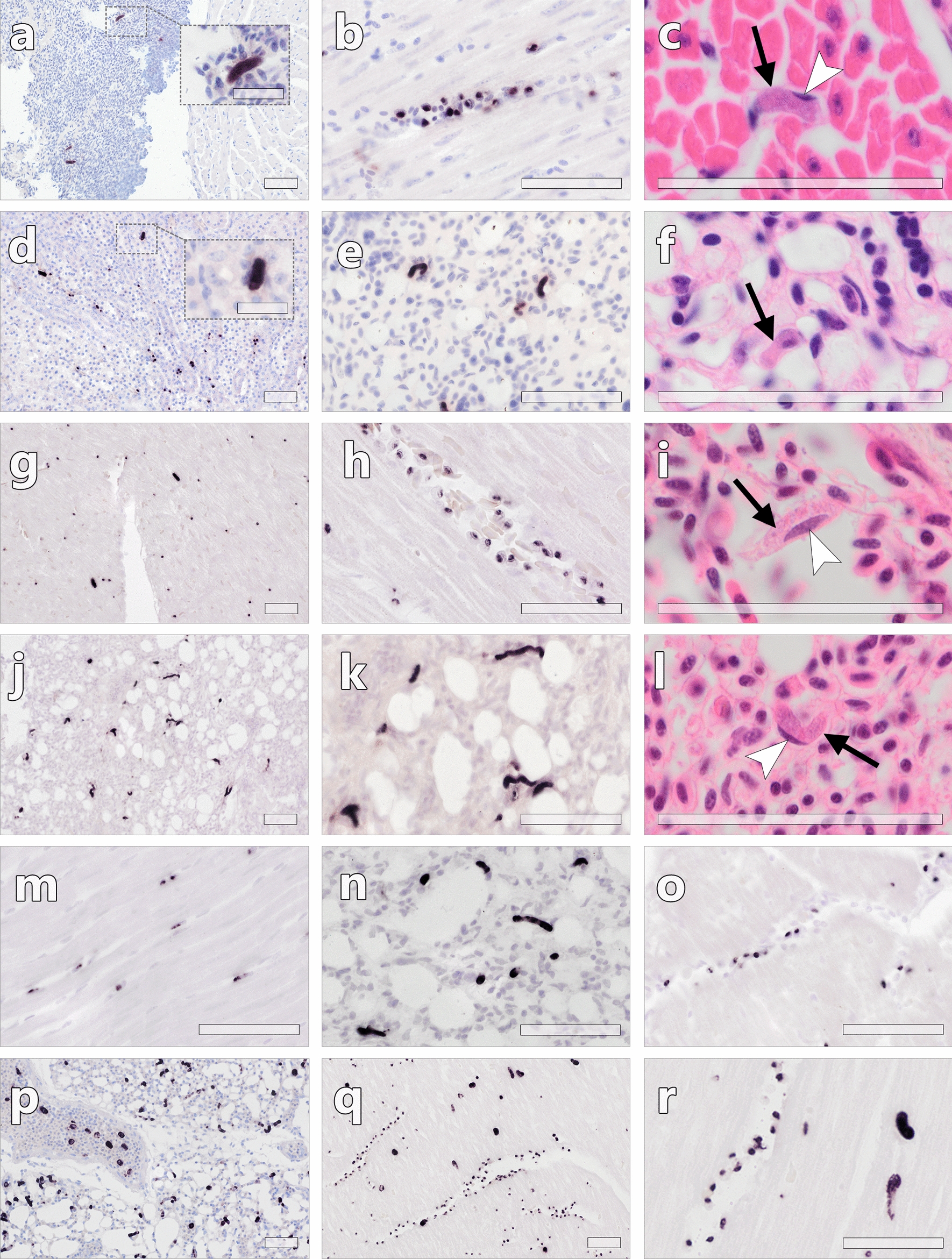
*Leucocytozoon* spp. blood stages (gametocytes) in histological sections of accipitriform birds (**a**–**f** *Accipiter nisus*, **g–l** *Circus aeruginosus*, **m**–**r** *Buteo buteo*). Blood stages were labelled by the *L. toddi*-specific probe during CISH (**a, b, d, e, g, h, j, k, m**–**r**) and identified in HE-stained sections (**c, f, i, l**). **a**–**f** Blood stages detected in capillaries and vessels in the heart (**a**–**c**), kidneys (**d**) and lungs (**e, f**) of an *Accipiter nisus* infected with *L.* aff*. toddi* lMILANS04. Note the presence of larger, elongate signals (**a**, **d,** arrowheads) besides numerous smaller roundish signals in the heart (**a, b**) and kidney (**d**). In the lung, the shape of the signals appeared wormlike (**e**). In HE-stained sections, elongate gametocytes were identified (**c, f,** black arrows), sometimes with a cap-like host cell nucleus visible (**c,** white arrowhead). **g**–**l** Blood stages detected in the heart (**g**–**i**) and lung (**j**–**l**) of a *Circus aeruginosus* infected with *L*. aff. *toddi* lCIAE03. Also in this individual, elongate (**g,** arrowheads) or wormlike (**j**, **k**) signals were observed alongside smaller roundish signals (**g, h**). In HE-stained sections, gametocytes were identified in fusiform host cells in heart vessels (**i**, black arrow) and in the lung (**l**, black arrow). Infected host cells showed an almond-shaped or cap-like nucleus (**i, l,** white arrowheads). **m**–**n** Blood stages detected in a *Buteo buteo* infected with *L*. aff. *toddi* lBUTBUT07. Blood stages detected in the heart were roundish to oval (**m**), while blood stages in the lung appeared wormlike (**n**). **o** Roundish blood stages detected in the heart of a *Buteo buteo* infected with *L*. aff. *toddi* lBUTBUT08. **p** Numerous oval to elongate blood stages detected in lung capillaries of a *Buteo buteo* infected with *L. buteonis* lBUBT2. **q**–**r** Blood stages detected in the heart of a *Buteo buteo* co-infected with *L. buteonis* lBUBT2 and *L*. aff. *toddi* lBUTBUT03. Blood stage signals were roundish and small or appeared large and elongate (arrowheads). Scale bars = 50 µm; insert scale bars = 20 µm

**Fig. 2:**
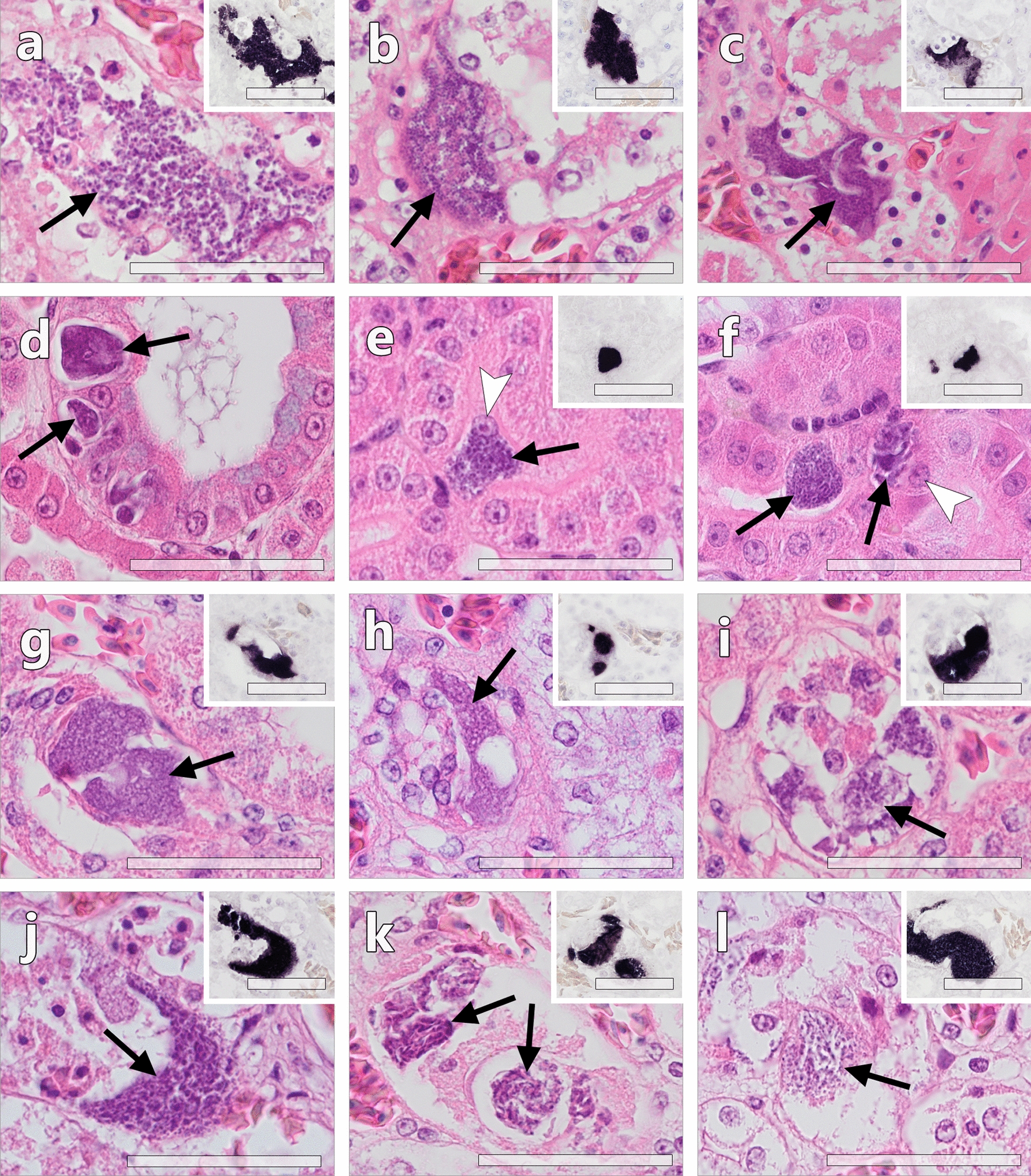
*Leucocytozoon* spp. tissue stages in kidney sections of accipitriform birds, detected by HE-staining and CISH (inserts). Meronts were frequently observed in epithelial cells of renal tubules and labelled by the *L. toddi*-specific probe (inserts, CISH). **a**–**d** Meronts (black arrows) detected in the kidneys of several *Buteo buteo* co-infected with *L. buteonis* lBUBT2 and *L.* aff*. toddi* lBUTBUT03. The meronts were of irregular shape and contained numerous basophilic merozoites. Several smaller meronts were located in renal epithelial cells (**d**). **e**–**f** Meronts detected in renal tubules of a *Buteo buteo* infected with *L. buteonis* lBUBT2. Mature meronts packed with merozoites often occupied the entire intracellular space of infected epithelial cells, causing protrusion into the tubular lumen and lateral displacement of the host cell nucleus (**e**, white arrow). **g**–**i** Meronts detected in renal tubules of a *Buteo buteo* infected with *L*. aff. *toddi* lBUTBUT12. Meronts shown in **g** and **h** contained nuclear material but developing merozoites were not discernible, indicating early stage of parasite development. Other meronts were more mature and contained developing merozoites (**i**). **j** A mature meront detected in a kidney tubule of a *Buteo buteo* co-infected with *L. buteonis* lBUBT2 and *L*. aff. *toddi* lBUTBUT07. **k**–**l** Developing meronts detected in a *Circus aeruginosus* co-infected with *L*. aff. *toddi* lCIAE03 and *P. circumflexum* pTURDUS1. Black arrow = meront, white arrowhead = host cell nucleus. All scale bars = 50 µm

Among 34 PCR-positive birds (with FFPE tissue samples available), 25 showed haemosporidian parasite stages in histological sections, albeit not of all recorded lineages (Table 3). Particularly *Plasmodium* parasites (*P. circumflexum*, *P. matutinum* and *P. elongatum*) were not detected by CISH in any of the birds confirmed positive for *Plasmodium* spp. by PCR. All 25 CISH-positive birds showed blood stages of the parasites (Fig. 1). In addition to blood stages, haemosporidian tissue stages were found in 14 birds, including 13 *Buteo buteo* infected with various *L. toddi* group lineages, and a *Circus aeruginosus* co-infected with *Leucocytozoon* sp. lCIAE03 and *P. circumflexum* pTURDUS1 (Table 3). Based on the labelling with the *L. toddi*-specific probe, most of the meronts could be attributed to lineages of the *L. toddi* species group. Meronts were observed exclusively in the kidneys, with an abundance ranging from single to a few meronts per renal cross-section. The meronts seemed to develop primarily in renal epithelial cells, although the exact location could not be determined for all of them due to poor preservation of some of the tissues. In some of the infected epithelial cells, a normal-sized host cell nucleus was visible (Fig. 2). Meronts ranged in size from approximately 10 µm to a maximum of 50 µm and varied in maturity not only between individuals but also in different organs of the same specimens. They contained more or less well-defined developing merozoites. Cytomeres could not be definitely distinguished. Megalomeronts were not observed in the inspected sections. As far as a histologic evaluation was possible, no major tissue alterations were associated with the detected tissue stages. In the *Circus aeruginosus* co-infected with pTURDUS1 and lCIAE03, a single meront of approximately 50 µm in length, was found in a HE-stained section of the heart muscle. However, due to the absence of this meront in the corresponding in situ hybridized section, its haemosporidian origin could not be confirmed by CISH. The morphology resembles tissue cysts of parasites belonging to the family Sarcocystidae (Conoidasida, Apicomplexa) (Additional file 1: Fig. S1).

**Table 3:** Haemosporidian parasite stages detected by CISH and histology in tissue sections of accipitriform birds

ID	Host species	Parasites	Blood stages^1,2^	Tissue stages (meronts)^1^
AH0817	*Accipiter gentilis*	*P. matutinum* pLINN1	−	−
AH0397	*Accipiter nisus*	*P. circumflexum* pTURDUS1	−	−
AH0810	*Accipiter nisus*	*L.* aff. *toddi* lMILANS04	+	−
AH1336	*Accipiter nisus*	*L. mathisi* lACNI04	−	−
AH1943	*Accipiter nisus*	*L.* aff. *toddi* lMILANS04	+	−
AH0156	*Buteo buteo*	*P.* cf. *circumflexum* pBT7	−	−
AH0182	*Buteo buteo*	*L. buteonis* lBUBT2, *L.* aff. *toddi* lBUTBUT07	+	Kidney
AH0234	*Buteo buteo*	*L. buteonis* lBUBT2	+	Kidney
AH0235	*Buteo buteo*	*L. buteonis* lBUBT2, *L.* aff. *toddi* lBUTBUT08	+	Kidney
AH0236	*Buteo buteo*	*L.* aff. *toddi* lBUTBUT03	+	−
AH0242	*Buteo buteo*	*L.* aff. *toddi* lBUTBUT07	+	Kidney
AH0244	*Buteo buteo*	*L. buteonis* lBUBT2, *L.* aff. *toddi* lBUTBUT03	+	Kidney
AH0252	*Buteo buteo*	*L.* aff. *toddi* lBUTBUT03, *L.* aff. *toddi* lBUTBUT13	+	−
AH0281	*Buteo buteo*	*L.* sp. lMILVUS02*, L.* aff. *toddi* lBUTBUT03, *P. circumflexum* pBT7	+	Kidney
AH0282	*Buteo buteo*	*L. buteonis* lBUBT2	−	−
AH0283	*Buteo buteo*	*L. buteonis* lBUBT2	+	−
AH0288	*Buteo buteo*	*L. buteonis* lBUBT2, *L.* aff. *toddi* lBUTBUT03	+	−
AH0291	*Buteo buteo*	*L. buteonis* lBUBT2, *L.* aff. *toddi* lBUTBUT03	+	Kidney
AH0293	*Buteo buteo*	*L. buteonis* lBUBT2	+	Kidney
AH0545	*Buteo buteo*	*L. buteonis* lBUBT2, *L.* aff. *toddi* lBUTBUT03	+	Kidney
AH0822	*Buteo buteo*	*L.* aff. *toddi* lBUTBUT03	+	−
AH1817	*Buteo buteo*	*L. buteonis* lBUBT2	+	−
AH1844	*Buteo buteo*	*L.* aff. *toddi* lBUTBUT08	+	Kidney
AH1863	*Buteo buteo*	*L. buteonis* lBUBT2, *L.* aff. *toddi* lBUTBUT09	+	Kidney
AH1920	*Buteo buteo*	*L. buteonis* lBUBT2	+	Kidney
AH1952	*Buteo buteo*	*L.* aff. *toddi* lBUTBUT12	+	Kidney
AH0249	*Circus aeruginosus*	*P.* cf. *elongatum* pMILANS05, *L.* aff. *toddi* lCIAE04	−	−
AH0250	*Circus aeruginosus*	*P. circumflexum* pTURDUS1, *L.* aff. *toddi* lCIAE03	+	Kidney
AH0254	*Circus aeruginosus*	*H.* sp. hCIAE08	+	−
AH0255	*Circus aeruginosus*	*L.* cf. *californicus* lCIAE02, *L. mathisi* lACNI04	+ (Leuco18S)	−
AH0260	*Circus aeruginosus*	*L.* cf. *californicus* lCIAE02, *L.* cf. *californicus* lCIAE06, *P. circumflexum* pTURDUS1	−	−
AH1869	*Circus aeruginosus*	*L. sp.* lASOT06, *L. buteonis* lBUBT2	−	−
AH1929	*Circus aeruginosus*	*L.* aff. *toddi* lCIAE03	+	−

^1^Unless otherwise stated, parasite stages were labelled with the *Leucocytozoon toddi*-specific probe (Ltod18S)

^2^−No blood stages detected, + blood stages detected

### Avian haemosporidian parasites in accipitriform raptors worldwide

#### *Leucocytozoon* parasites in accipitriform raptors

**Fig. 3:**
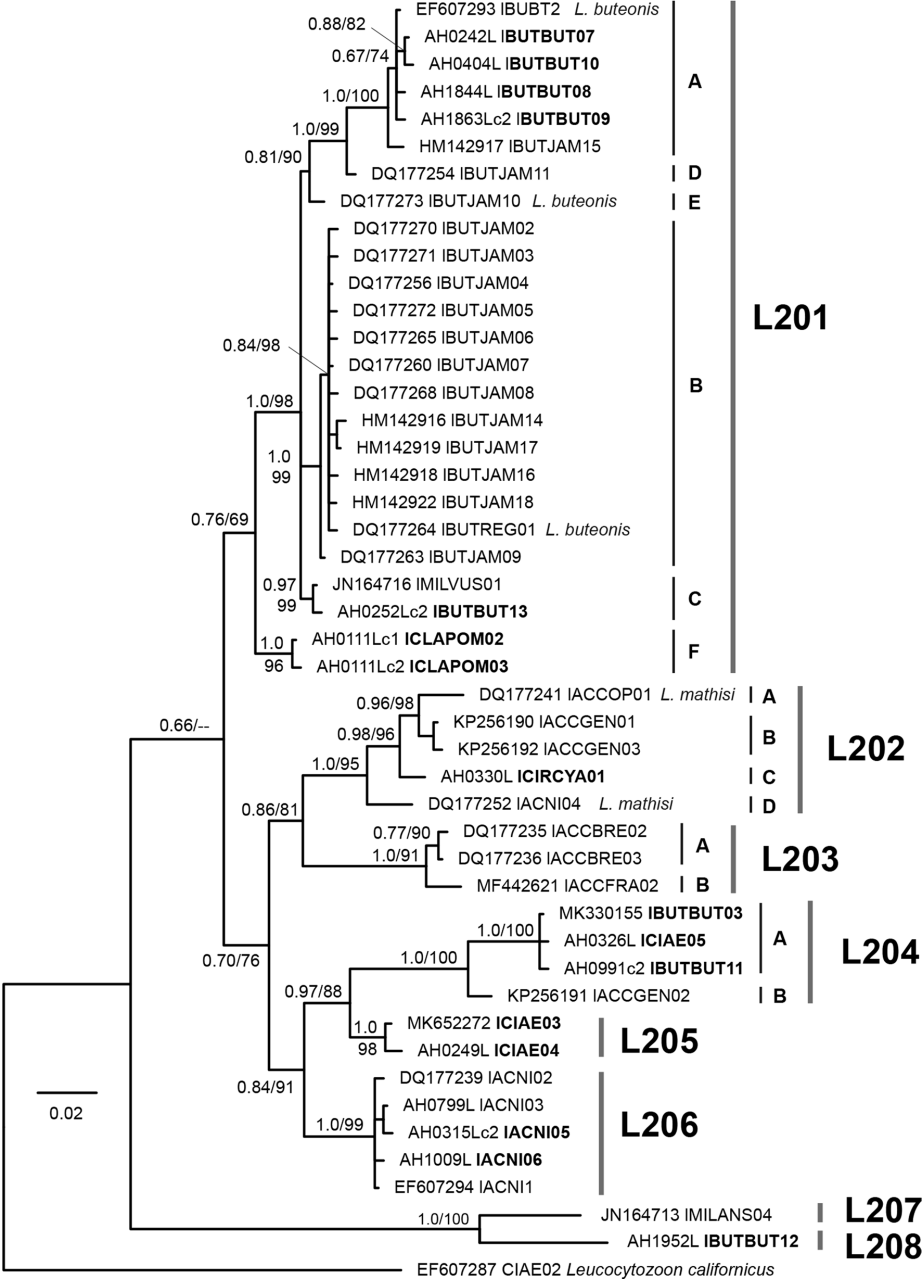
Phylogeny of haemosporidian *CytB* lineages (474 bp) belonging to the *Leucocytozoon toddi* species group. Bayesian posterior probabilities and Maximum likelihood bootstrap values are indicated at most nodes. The scale bars indicate the expected number of substitutions per site according to the model of sequence evolution applied. Bold letters indicate lineage names found for the first time in the present study. Species names indicate that the lineages were already linked to the respective morphospecies

All 10 *Leucocytozoon* parasites described from accipitriform hosts belong to the *L. toddi* species group, but most names were previously synonymized with *L. toddi* due to morphological similarity of their gametocytes and host-cells [3]. However, the genetic diversity within this clade is high, potentially indicating a higher number of parasite species than previously assumed. The only other clade featuring multiple *Leucocytozoon* lineages from accipitriform raptors contains the sequence linked to *L. californicus* and related parasite lineages. Another five lineages clustered into different clades, four of which were found mostly in birds of other orders and probably appeared in accipitriform birds as abortive infections. Table 4 features a summary of *Leucocytozoon* lineages found in accipitriform raptors. Two separate BI trees are provided for the *L. toddi* group (Fig. 3) and the other *Leucocytozoon* lineages (Additional file 2: Fig. S2).

**Table 4:** *CytB* lineages of *Leucocytozoon* spp. in accipitriform raptors

Lineage	Accession	Parasite species	Host species	Host environment	Countries	References
*Leucocytozoon toddi* group						
lBUBT2	EF607293	*L. buteonis*	*Buteo buteo* (27), *Accipiter gentilis* (1), *Circus aeruginosus* (1)	Wild/captive	Austria, B.-H., Kazakhstan	[9]; present study
lBUTBUT07	OL598481	*L.* sp.	*Buteo buteo* (5)*, Buteo lagopus* (1)	Wild	Austria	Present study
lBUTBUT08	OL598478	*L.* sp.	*Buteo buteo* (2)	Wild	Austria	Present study
lBUTBUT09	OL598480	*L.* sp.	*Buteo buteo* (1)	Captive	Austria	Present study
lBUTBUT10	OL598487	*L. buteonis*	*Buteo buteo* (1)	Wild	Austria	Present study
lBUTJAM15	HM142917	*L.* sp.	*Buteo jamaicensis* (1)	Wild	USA	[10]
lBUTJAM04	DQ177269	*L.* sp.	*Buteo jamaicensis* (22)	Wild	USA	[9, 10]
lBUTJAM07	DQ177255	*L.* sp.	*Buteo jamaicensis* (16), *Buteo lineatus* (2)	Wild	USA	[9, 10]
lBUTREG01	DQ177264	*L. buteonis*	*Buteo jamaicensis* (1), *Buteo regalis* (1)	Wild	USA	[9, 10]
lBUTJAM02	DQ177270	*L.* sp.	*Buteo jamaicensis* (1)	Wild	USA	[9]
lBUTJAM03	DQ177271	*L.* sp.	*Buteo jamaicensis* (1)	Wild	USA	[9]
lBUTJAM05	DQ177272	*L.* sp.	*Buteo jamaicensis* (1)	Wild	USA	[9]
lBUTJAM06	DQ177265	*L.* sp.	*Buteo jamaicensis* (1)	Wild	USA	[9]
lBUTJAM08	DQ177268	*L.* sp.	*Buteo jamaicensis* (1)	Wild	USA	[9]
lBUTJAM09	DQ177263	*L.* sp.	*Buteo jamaicensis* (1)	Wild	USA	[9]
lBUTJAM14	HM142916	*L.* sp.	*Buteo jamaicensis* (1)	Wild	USA	[10]
lBUTJAM16	HM142918	*L.* sp.	*Buteo jamaicensis* (1)	Wild	USA	[10]
lBUTJAM17	HM142919	*L.* sp.	*Buteo jamaicensis* (1)	Wild	USA	[10]
lBUTJAM18	HM142922	*L.* sp.	*Buteo jamaicensis* (1)	Wild	USA	[10]
lMILVUS01	JN164716	*L.* sp.	*Buteo buteo* (4), *Milvus milvus* (4), *Milvus migrans* (1)	Wild/captive	Austria, Spain	[8]; present study
lBUTBUT13	OL598492	*L.* sp.	*Buteo buteo* (1)	Wild	Austria	Present study
lBUTJAM11	DQ177254	*L.* sp.	*Buteo jamaicensis* (1)	Wild	USA	[9]
lBUTJAM10	DQ177273	*L.* sp.	*Buteo jamaicensis* (1)	Wild	USA	[9]
lCLAPOM02	OL598493	*L.* sp.	*Clanga pomarina* (1)	Wild	Austria	Present study
lCLAPOM03	OL598494	*L.* sp.	*Clanga pomarina* (1)	Wild	Austria	Present study
lACCOP01	DQ177241	*L. mathisi*	*Accipiter cooperi* (11)	Wild	USA	[9]
lACCGEN01	KP256190	*L.* sp.	*Accipiter gentilis* (11)	Wild	Czechia, Austria	[12]; Present study
lACCGEN03	KP256192	*L.* sp.	*Accipiter gentilis* (1)	Wild	Czechia	[12]
lCIRCYA01	OL598499	*L.* sp.	*Circus cyanaeus* (1)	Wild	Austria	Present study
lACNI04	DQ177252	*L. mathisi*	*Accipiter nisus* (3), *Circus aeruginosus* (1)	Wild	Austria, Kazakhstan	[9]; Present study
lACCBRE02	DQ177235	*L.* sp.	*Accipiter brevipes* (1)	Wild	Kazakhstan	[9]
lACCBRE03	DQ177236	*L.* sp.	*Accipiter brevipes* (1)	Wild	Kazakhstan	[9]
lACCFRA02	MF442621	*L.* sp.	*Accipiter francesiae* (1)*, Accipiter madagascariensis* (1)	Wild	Madagascar	[30]
lBUTBUT03	MK652270	*L.* sp.	*Buteo buteo* (13), *Accipiter gentilis* (2)	Wild/captive	Austria	Present study
lBUTBUT11	OL598440	*L.* sp.	*Buteo buteo* (1)	Captive	Austria	Present study
lCIAE05	OL598441	*L.* sp.	*Circus aeruginosus* (1)	Wild	Austria	Present study
lACCGEN02	KP256191	*L.* sp.	*Accipiter gentilis* (1)	Wild	Czechia	[12]
lCIAE03	MK652272	*L.* sp.	*Circus aeruginosus* (5)	Wild/captive	Austria	Present study
lCIAE04	OL598446	*L.* sp.	*Circus aeruginosus* (1)	Wild	Austria	Present study
lACNI01	MF928785	*L.* sp.	*Accipiter nisus* (4)	Wild/captive	Austria, Germany, Kazakhstan, Turkey	[14, 147]; Simsek et al. unpub.; present study
lACNI02	DQ177239	*L.* sp.	*Accipiter nisus* (2)	Wild	Kazakhstan	[9]
lACNI03	DQ177237	*L.* sp.	*Accipiter nisus* (3)	Wild	Austria, Kazakhstan, Turkey	[9]; Simsek et al. unpub.; present study
lACNI05	OL598447	*L.* sp.	*Accipiter nisus* (1)	Wild	Austria	Present study
lACNI06	OL598448	*L.* sp.	*Accipiter nisus* (1)	Captive	Austria	Present study
lACNI07	OL598450	*L.* sp.	*Accipiter nisus* (1)	Wild	B.-H	Present study
lACNI02	DQ177239	*L.* sp.	*Accipiter nisus* (1)	Wild	Kazakhstan	[9]
lMILANS04	JN164713	*L.* sp.	*Milvus milvus* (3)*, Milvus migrans* (1)*, Accipiter nisus* (2)	Wild/captive	Spain, Austria	[8]; present study
lBUTBUT12	OL598531	*L.* sp.	*Buteo buteo* (1)	Captive	Austria	Present study
*Leucocytozoon californicus* group						
lCIAE02	EF607287	*L.* aff. californicus	*Accipiter nisus* (3)*, Aquila heliaca* (1)*, Buteo buteo* (2)*, Buteo rufinus* (1)*, Circus aeruginosus* (6)*, Circus cyaneus* (1)*, Milvus migrans* (16)		Austria, China, Germany, Spain, Thailand, Turkey	[8, 11, 14, 15, 69]; Present study
lBUTBUT01	KP000841	*L.* aff. californicus	*Buteo buteo* (1)	Wild	Turkey	[15]
lCIAE06	OL598524	*L.* aff. californicus	*Circus aeruginosus* (1)	Wild	Austria	Present study
ACCTRI01	KX950744	*L.* aff. californicus	*Accipiter trivergatus* (1)	Unknown	Thailand	Prasopsom et al*.* unpub
Other *Leucocytozoon* lineages						
lBT2	AY393802	*L.* sp.	*Accipiter gentilis* (2)	Wild	Czechia	[12]
lBUTBUT05	MT281506	*L.* sp.	*Buteo buteo* (1)	Captive	China	[11]
lBUBO01	KF146934	*L. danilewskyi*	*Buteo buteo* (1)		Iran	[50]
lASOT06	MT281492	*L.* sp.	*Accipiter nisus* (3)	Captive	Austria, China	[11]; present study
lMILVUS02	JN164717	*L.* sp.	*Milvus milvus* (1), *Buteo buteo* (1), *Buteo lagopus* (1), *Haliaeetus albicilla* (1)	Wild	Austria, Spain	[8]; present study

The numbers in the brackets indicate the number of individuals that featured the respective lineages. One GenBank accession number is indicated for each MalAvi lineage. The term ‘aff.’ (‘species affinis’) indicates that the lineage is similar to other lineages, which were already linked to morphospecies

#### *Leucocytozoon toddi* species group

Parasite lineages of the *L. toddi* species group were exclusively found in accipitriform raptors and belong to a clade, which differs from the other *Leucocytozoon* taxa by about 20% in the *CytB*. The lineages were found in birds in Europe, Northern America and Western Asia.

**Fig. 4:**
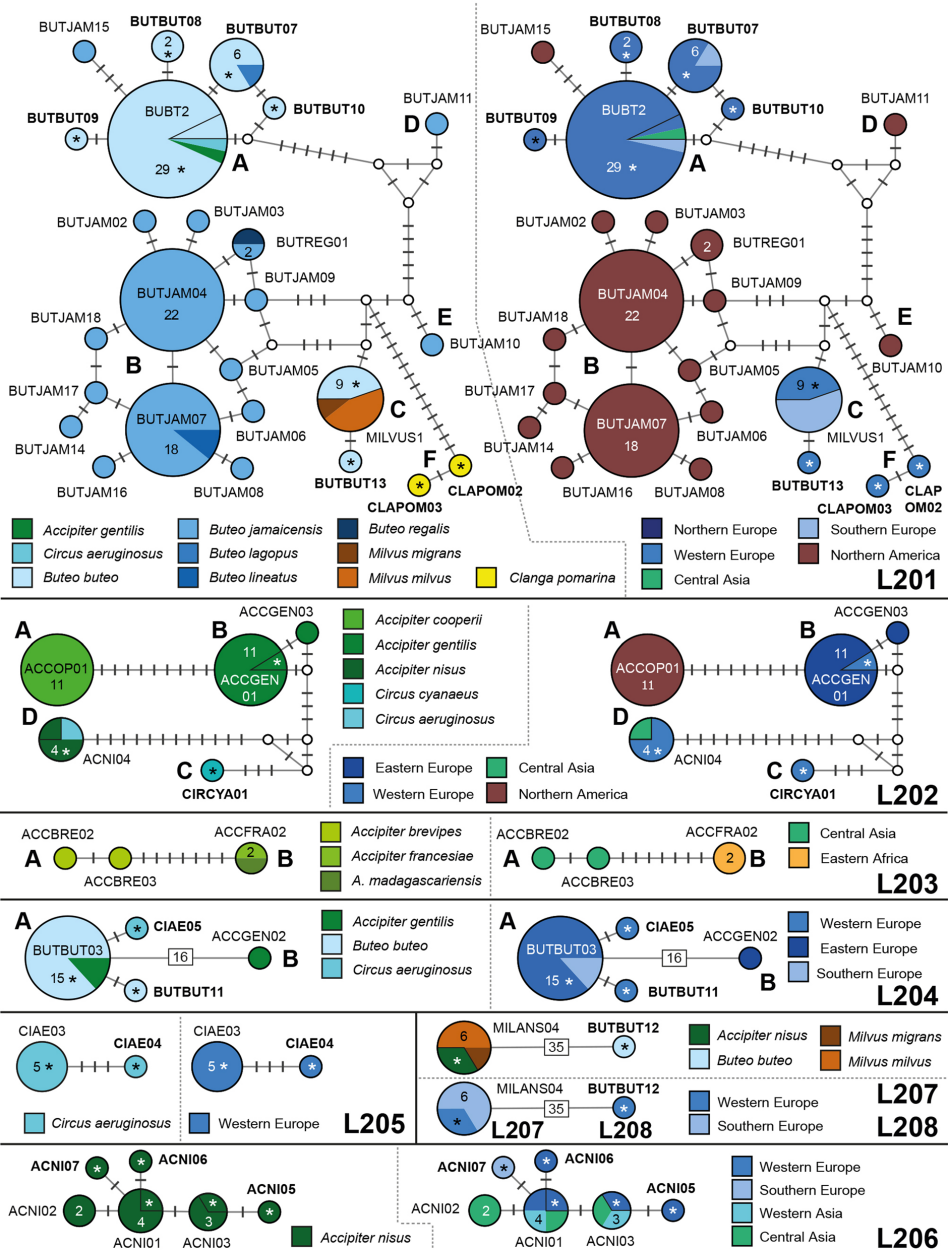
Median-Joining DNA haplotype network of partial (474 bp) *CytB* sequences belonging to the *Leucocytozoon toddi* species group. Two representations were prepared for each network, the first indicating the host species and the second the geographic origin according to the United Nations geoscheme. Asterisks mark haplotypes detected in the present study and new lineage names are indicated in bold letters. Each circle represents a unique haplotype/lineage. The frequency of each lineage is indicated for all haplotypes with more than one record and corresponds to the size of circles. Bars on branches and numbers in squares indicate the number of substitutions between two haplotypes. Small white circles represent median vectors, which are hypothetical (often ancestral or unsampled) sequences required to connect existing haplotypes with maximum parsimony

Eight major clades (L201 to L208; Fig. 3), separated by more than 5.6% *p*-distance in the *CytB* barcode section, were identified. These clades feature 18 sub-clades separated by at least five bp (ca 1.2%) from each other. The haplotypes/lineages within the sub-clades differ by three bp or less from each other or the most common, central haplotypes. Haplotype networks showing the geographic and host distribution of the lineages within each clade are shown in Fig. 4.

Clade L201 contains five sub-clades featuring similar lineages (L201a to L201f). Sub-clade L201a contains lBUBT2 (29), lBUTBUT07 (6), lBUTBUT08 (2), lBUTBUT09 (1), lBUTBUT10 (1), and lBUTJAM15 (1). Lineage lBUTJAM15 was found in *Buteo jamaicensis* (1) in the USA [10], the others mostly in *Buteo buteo* in Western Europe. Lineage lBUBT2 was found in *Buteo buteo* from Austria (24), B.-H. (1) and Kazakhstan (1), and in *Accipiter gentilis* (1) and *Circus aeruginosus* (1) from Austria [9, 14]. Lineage lBUTBUT07 was found in *Buteo buteo* from Austria (4) and B.-H. (1), and in *Buteo lagopus* (1) from Austria. The lineages lBUTBUT08 (2), lBUTBUT09 (1), and lBUTBUT10 (1) were exclusively detected in *Buteo buteo* in Austria. Sub-clade L201b contains lBUTJAM04 (22), lBUTJAM07 (18), lBUTREG01 (2), lBUTJAM02 (1), lBUTJAM03 (1), lBUTJAM05 (1), lBUTJAM06 (1), lBUTJAM08 (1), lBUTJAM09 (1), lBUTJAM14 (1), lBUTJAM16 (1), lBUTJAM17 (1), and lBUTJAM18 (1) [9, 10]. All lineages were exclusively found in *Buteo jamaicensis* in the USA, except for lBUTREG01 and lBUTJAM07, which were also found in single individuals of *Buteo regalis* and in *Buteo lineatus*, respectively. Sub-clade L201c features lMILVUS01 (9) and lBUTBUT13 (1). Lineage lMILVUS01 was found in *Buteo buteo* (4) in Austria and *Milvus milvus* (4) and *Milvus migrans* (1) in Spain [8], and lBUTBUT13 was detected in a single specimen of *Buteo buteo* in Austria. Sub-clades L201d (lBUTJAM11) and L201e (lBUTJAM10) feature single lineages reported from *Buteo jamaicensis* in the USA [9, 10]. Last, sub-clade L201f contains lCLAPOM02 and lCLAPOM03, which were detected in one specimen of the lesser spotted eagle *Clanga pomarina* in Austria. Based on a combined analysis of morphological and *CytB* sequence data, [29] linked lBUBT2 (L201a), lBUTJAM10 (L201e), and lBUTREG01 (L201b) to *Leucocytozoon buteonis*. The latter three lineages belong to distinct sub-clades (L201, Fig. 4) and differ by 1.5 to 3.4% from each other in the *CytB*. The patterns regarding host and geographic distribution suggest that the lineages in clade L201 might belong to six different parasite species.

Clade L202 features five lineages in four sub-clades. L202a contains lACCOP01, exclusively detected in *Accipiter cooperi* (11) in the USA [9]. L202b contains lACCGEN01 (11) and lACCGEN03 (1), exclusively found in *Accipiter gentilis* from Czechia [12] and Austria. L202c features lCIRCYA01 from *Circus cyanaeus* (1) in Austria. Last, sub-clade L202d contains lACNI04 found in *Accipiter nisus* (3) from Austria and Kazakhstan [9] and in *Circus aeruginosus* (1) from Austria. Valkiūnas et al*.* [29] linked lACCOP01 and lACNI04 to *Leucocytozoon mathisi* based on the morphological similarity of their gametocytes and host cells. The two latter lineages fall into sub-clades L201a and L201b and differ by 4.6% in the *CytB*. The genetic distances between lineages, the observed host specificity, and their geographic distribution suggests that the five lineages potentially belong to four distinct parasite species of *L. mathisi*.

Clade L203 features three lineages in two sub-clades. Sub-clade L203a contains lACCBRE02 and lACCBRE03, each found in single individuals of *Accipiter brevipes* in Kazakhstan [9]. Sub-clade L203b only features lACCFRA02 found in *Accipiter francesiae* (1) and *Accipiter madagascariensis* (1) from Madagascar [30].

Clade L204 features four lineages in two sub-clades. Sub-clade L204a contains lBUTBUT03 from *Buteo buteo* (13) in Austria and *Accipiter gentilis* (2) in B.-H., lBUTBUT11 from *Buteo buteo* (1) in Austria, and lCIAE05 from *Circus aeruginosus* (1) in Austria. Sub-clade L204b contains only lACCGEN02 from one individual of *Accipiter gentilis* sampled in Czechia [12].

Clade L205 contains lCIAE03 (5) and lCIAE04 (1), which were exclusively detected in *Circus aeruginosus* in Austria.

Clade L206 contains six lineages exclusively found in *Accipiter nisus*. Lineage lACNI01 was found in Austria (1), Germany (1) [14], Kazakhstan (1) [9], and Turkey (1) (Simsek et al. unpublished), and lACNI03 was found in Austria (1), Kazakhstan (1) [9], and Turkey (1) (Simsek et al. unpublished). Lineages lACNI05 (1) and lACNI06 (1) were found in Austria, lACNI07 (1) in B.-H., and lACNI02 (2) in Kazakhstan [9]. The lineages within clade L206 are similar and were found only in *Accipiter nisus*, suggesting that they belong to a *Leucocytozoon* parasite specific to this host species. The data also suggest that at least two species of this parasite group might infect *Accipiter nisus*, *L. mathisi*, and another *L. toddi* group parasite.

Clade L207 and L208 together form a clade taking the most basal position in the phylogeny of the *L. toddi* species group (Fig. 4), differing by more than 15% in the *CytB* from the other clades. Clade L207 features lineage lMILANS04, which was found in *Milvus milvus* (3) and *Milvus migrans* (1) from Spain [8], and in *Accipiter nisus* (2) from Austria. Clade L208 features lineage lBUTBUT12, found in one specimen of *Buteo buteo* in Austria.

#### *Leucocytozoon californicus *species group

**Fig. 5:**
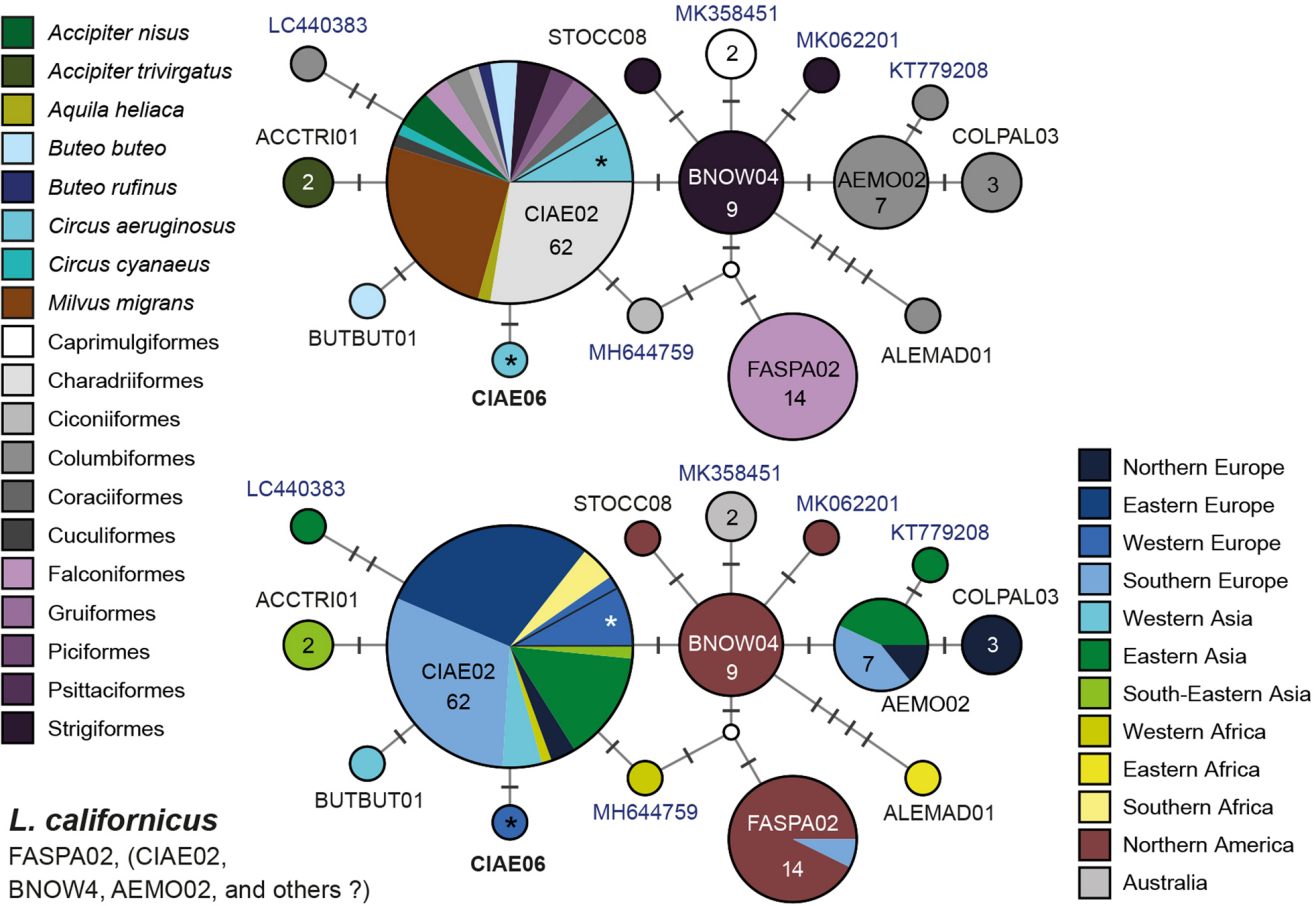
Median-Joining DNA haplotype network of partial (474 bp) *CytB* sequences belonging to the *Leucocytozoon californicus* species group. The only lineage linked to *L. californicus* so far is lFASPA02. The upper image indicates the number and frequency of host species, the lower one the geographic origin according to the United Nations geoscheme. Asterisks mark haplotypes detected in the present study. New lineage names are indicated in bold letters. In case lineages were not listed in the MalAvi database, the GenBank accession numbers are indicated in blue

*Leucocytozoon californicus* was described from *Falco sparverius* in California (USA) and linked to lineage lFASPA02 by [31]. Morphologically similar parasites were found before in migrating falconiform birds in Southern Kazakhstan, indicating that transmission of *L. californicus* might also take place in Eurasia [20, 31]. The lineage was later found in *Falco columbarius* from Italy, confirming its presence in Europe [32]. Lineage lFASPA02 is part of a clade (Fig. 5), which features 15 lineages found in Accipitriformes, Falconiformes, Strigiformes, and members of other bird orders. The most frequent lineage lCIAE02 was found in 62 birds of the orders Accipitriformes (30), Charadriiformes (17), Strigiformes (3), Falconiformes (2), Columbiformes (2), Coraciiformes (2), Gruiformes (2), Piciformes (2), Cuculiformes (1), and Ciconiiformes (1) in Europe, Asia and Africa (Additional file 3: Table S1). In accipitriform raptors, lCIAE02 was detected in *Accipiter nisus* from China (3) [11], *Aquila heliaca* from Thailand (1) [33], *Buteo buteo* from Turkey (1) [15] and China (1) [11], *Buteo rufinus* from Turkey (1) [15], *Circus aeruginosus* from Austria (5) and Germany (1) [14], *Circus cyaneus* from China (1) [11], and *Milvus migrans* from Spain (16) [8]. Lineage lBNOW04 was exclusively reported from Strigiformes (9) in the USA [34, 35] and lFASPA02 from Falconiformes in the USA (13) [31] and Italy (1) [32]. Lineage lAEMO02 was found in Columbidae in Japan (2) [36], Taiwan (1) (Huang et al., unpublished; KT779209), Spain (1) [37], Portugal (2) [38, 39], and the UK (1) [40]. The clade contains several less frequent lineages, which are separated from the latter ones by one to three bp: lBUTBUT01 from *Buteo buteo* (1) in Turkey [15], lCIAE06 from *Circus aeruginosus* (1) in Austria, lACCTRI01 from *Accipiter trivergatus* (2) in Thailand [41], lALEMAD01 from the Madagascan blue pigeon *Alectroenas madagascariensis* (1) in Madagascar [30], lCOLPAL03 from the common wood pigeon *Columba palumbus* (3) in the UK [40], MH644759 from the western cattle egret *Bubulcus ibis* (1) in Western Africa [42], KT779208 from the red collared dove *Streptopelia tranquebarica* (1) in Taiwan (Huang Y. L. et al., unpublished), MK062201 from the northern saw-whet owl *Aegolius acadicus* (1) in the USA [34], MK358451 from the tawny frogmouth *Podargus strigoides* (2) in Australia [43], and LC440383 from the whistling green pigeon *Treron formosae* (1) in Japan [44]. The *L. californicus* clade is exceptional because it contains several closely related lineages from birds of various host orders and geographic regions. While the other lineages were each found in birds of one host order, lCIAE02 was found in birds belonging to 10 different host orders from Eurasia and Africa. Further studies on lCIAE02 and other lineages are required to confirm or refute their identity as *L. californicus*.

#### Other *Leucocytozoon* lineages in accipitriform raptors

Another five *Leucocytozoon* lineages were found in a few accipitriform birds. Lineage lBT2 was detected in two wild juvenile *Accipiter gentilis* from Czechia [12]. The lineage was found in more than 100 passeriform birds (mainly Sylviidae, Muscicapidae, and Fringillidae) in Europe, China, and Nigeria [38, 45, 46]. It was also found in both juvenile and adult boreal owls *Aegolius funereus* (7) in Czechia [47].

Lineage lBUTBUT05 was detected in *Buteo buteo* (1) kept at the Beijing Raptor Rescue Centre in Beijing, China [11]. Elsewhere, the lineage was found in blood-engorged females of the black fly species *Simulium chumpornense* in Thailand. Blood meal analysis revealed that the black flies were feeding on domestic chicken *Gallus gallus* [48]. Lineage lBUTBUT05 is similar to more than 20 haemosporidian yet unnamed lineages, which were found in *Simulium asakoae* and other black flies in Thailand [49].

Lineage lBUBO01 was found in *Buteo buteo* (1) and the Eurasian eagle-owl *Bubo bubo* (1) kept together at a rehabilitation centre in Mashhad, Iran [50], and in *Bubo bubo* in Austria (2) [23] and Spain (1) [51]. Ortego & Cordero [51] identified the lineage as *Leucocytozoon ziemanni*, which is a synonym of *Leucocytozoon danilewskyi*, a common parasite in owls [3].

Lineage lASOT06 was found in *Accipiter nisus* (1) and *Circus aeruginosus* (1) from Austria, and *Accipiter nisus* (1) from China [11]. It was detected in the owls *Asio otus* (6), *Athene noctua* (2), and *Aegolius funereus* (1) in China [11], *Asio otus* (1) in Germany [14], *Falco tinnunculus* (1) in China [11], and the common blackbird *Turdus merula* (4) in Austria [1, 4]. The three accipitriform raptors infected with lASOT06 were kept at rehabilitation facilities together with owls, which are probably the natural hosts of this lineage. The CISH performed on the *Circus aeruginosus* sample was negative, which was also the case for *Turdus merula* investigated by [1], thus indicating that both species are probably not competent hosts.

Lineage lMILVUS02 was found in *Buteo buteo* (1), *Buteo lagopus* (1) and *Haliaeetus albicilla* (1) from Austria (present study), and *Milvus milvus* (1) from Spain [8]. The lineage resembles lFURRUF01 (8 bp difference) from the rufous hornero *Furnarius rufus* and lPHARUB01 (9 bp difference) from the sepia-capped flycatcher *Leptopogon amaurocephalus* (1) and the reddish hermit *Phaethornis ruber* (1), which were all sampled in Brazil [52].

### *Plasmodium* parasites in accipitriform raptors

Only one of the known *Plasmodium* species, *Plasmodium accipiteris*, has been described specifically from accipitriform raptors, but three species were reported to also infect birds of this host group: *P. circumflexum*, *P. fallax*, and *P. forresteri* [3, 53]. Of these, *P. circumflexum* is the only *Plasmodium* species, which was found in more than two species of accipitriform hosts and which was already characterized by DNA barcoding. Three clades were identified, which feature several lineages detected in accipitriform hosts. None of these lineages could be linked to any known morphospecies. *Plasmodium fallax* and *P. forresteri* are only known from morphological and experimental studies, but the *CytB* lineages of these species have not been identified yet. In total, 25 *Plasmodium* lineages were reported from accipitriform birds (Table 5). A BI tree featuring all *Plasmodium* lineages from accipitriform raptors is shown in Additional file 4: Fig. S3.

**Table 5:** *Plasmodium CytB* lineages found in accipitriform raptors

Lineage	Accession	Parasite species	Host species (only accipitriform birds)	Host env	Countries	References
pTURDUS1	AF495576	*P. circumflexum*	*Accipiter nisus* (8)*, Accipiter gularis* (3)*, Accipiter gentilis* (2)*, Buteo buteo* (8)*, Circus aeruginosus* (5)	Wild/captive	Austria, B.-H., China, Czechia, Germany, Japan, Sweden, Turkey	[11, 12, 14, 15, 38, 44]; present study
pBT7	AY393793	*P. circumflexum*	*Buteo buteo* (3)*, Buteo lagopus* (1)*, Buteo rufinus* (1)*, Accipiter nisus* (2)*, Accipiter gentilis* (1)*, Accipiter gularis* (1)*, Accipiter striatus* (1)	Wild/captive	Austria, China, Germany, Iran, USA	[11, 14, 58]; present study
pCIAE07	OL598513	*P.* aff. *circumflexum*	*Circus aeruginosus* (1)	Captive	Austria	present study
pBUTBUT02	KP883279	*P.* aff. *circumflexum*	*Accipiter nisus* (1)*, Buteo buteo* (1)	Wild	Turkey	[15]
pLINN1	DQ847270	*P. matutinum*	*Accipiter gentilis* (1)	Captive	Austria	present study
pMYCAME02	JX546135	*P. paranucleophilum*	*Buteo brachyurus* (1)*, Rupornis magnirostris* (1)	Captive	Brazil	[88]
pEMSPO06	EF380135	*P.* aff. *homonucleophilum*	*Accipiter nisus* (1)	Captive	China	[11]
pMILANS05	JN164714	*P.* aff. *elongatum*	*Circus aeruginosus* (1)	Wild	Austria	present study
pPLACAS02	EU810612	*P.* aff. *elongatum*	*Accipiter tachiro* (1)	Wild	Gabon	[64]
pACCBAD01	JN639001	*P.* sp.	*Accipiter badius* (2)	Captive	Thailand	[68]
pACCNIS05	MT281522	*P.* sp.	*Accipiter nisus* (1)	Captive	China	[11]
pBUTBUT06	MT281514	*P.* aff. *gallinaceum*	*Buteo buteo* (1)	Captive	China	[11]
pMILANS06	JN164715	*P.* sp.	*Milvus migrans* (1)	Wild	Spain	[8]
pNYCNYC01	KU057967	*P.* sp.	*Sarcoramphus papa* (2)	Captive	Brazil	[87]
pPESA01	EU684543	*P.* sp.	*Buteogallus urubitinga* (1)	Captive	Brazil	[70]
pRHYSIM01	KU562769	*P.* sp.	*Nisaetus alboniger* (1)	Uncertain	Thailand	[92]
pRTSR1	AF495568	*P.* sp.	*Aquila wahlbergi* (1)	Wild	Malawi	[95]
pSYBOR10	DQ368390	*P.* sp.	*Circus aeruginosus (1)*	Wild	Austria	present study
pZEMAC01	AY099032	*P.* sp.	*Aquila chrysaetos* (2)	Captive	USA	[100]
pORW1	AF254963	*P.* sp.	*Gyps bengalensis* (14)	Wild	India	[13]
pACCTAC01	EU810700	*P.* sp.	*Accipiter tachiro (1)*	Wild	Gabon	[64]
pGYPTEN01	DQ212194	*P.* sp.	*Gyps tenuirostris* (1)	Wild	Cambodia	Duval et al. unpub
pGYPBEN01	DQ212195	*P.* sp.	*Gyps bengalis* (1)	Wild	Cambodia	Duval et al. unpub
pCIAE01	EF607288	*P.* sp.	*Circus aeruginosus* (1)	Wild	Germany	[14]
pHALVOC01	EF011195	*P.* sp.	*Haliaeetus vocifer* (1)	Wild	Uganda	[67]

The list features records of all *Plasmodium CytB* lineages found in accipitriform birds, other host species are not listed. The numbers in the brackets indicate the number of individuals that featured the respective lineages. One GenBank accession number is indicated for each MalAvi lineage. The term ‘aff.’ means ‘species affinis’, indicating that the lineage is similar to others, which were already linked to morphospecies

#### *Plasmodium* clade 1: *Plasmodium circumflexum*

**Fig. 6:**
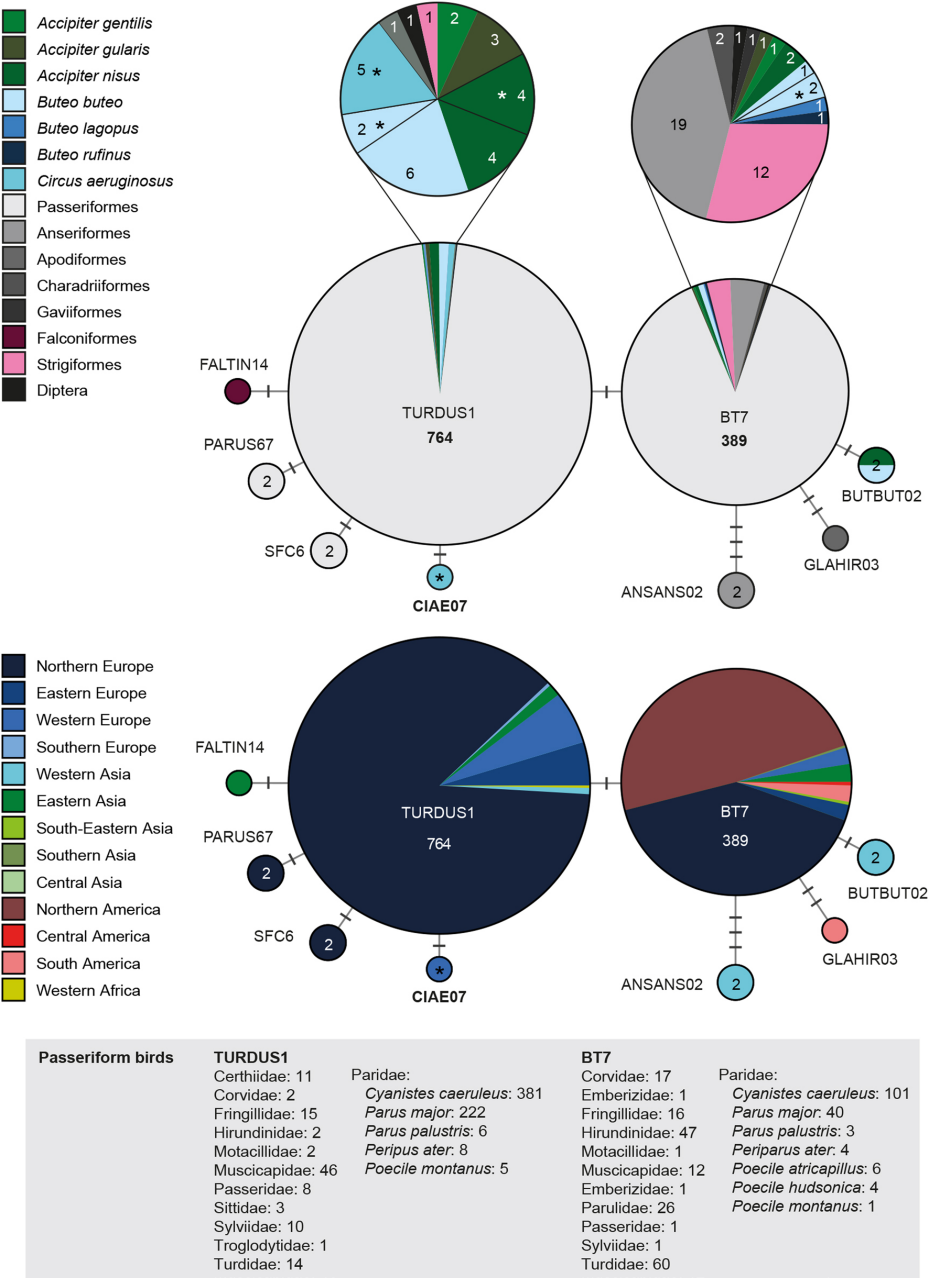
Median-Joining DNA haplotype network of partial (474 bp) *CytB* sequences belonging to the *Plasmodium circumflexum* species group. The upper image indicates the number and frequency of host species, the lower one the geographic origin according to the United Nations geoscheme. Asterisks mark haplotypes detected in the present study. New lineage names are indicated in bold letters

Palinauskas et al. [54] linked the lineage pTURDUS1 to *P. circumflexum* and suggested that pBT7, which differs in one bp from pTURDUS1 in the *CytB*, might belong to the same morphospecies. These two common lineages are part of one sub-clade together with seven rare and closely related lineages (Fig. 6). The entire clade features several additional, less closely related lineages (see Discussion), which might resemble *P. circumflexum* morphologically as well.

Most records of pTURDUS1 (764 records in total) originate from Northern Europe (662), Western Europe (42) and Eastern Europe (35), whereas the lineage was rarely recorded in Eastern Asia (10), Western Asia (6), Southern Europe (4), and Western Africa (2), and has never been found in the Americas so far. The most common hosts of pTURDUS1 are birds of the families Paridae (620), Muscicapidae (45), Turdidae (14), Fringillidae (14), Certhiidae (11), and Sylviidae (10). The two Paridae species *Cyanistes caeruleus* (381) and *Parus major* (222) together constitute more than 80% of all pTURDUS1 records. Although only 26 records originate from accipitriform raptors, pTURDUS1 is still one of the most common haemosporidian lineages in this host group and was found in *Accipiter nisus* (8), *Buteo buteo* (8), *Circus aeruginosus* (5), *Accipiter gularis* (3), and *Accipiter gentilis* (2) [11, 12, 14, 15, 38, 44]. There is only one record of pTURDUS1 from Strigiformes (*Aegolius funereus*; [47]), but no records from Falconiformes, Anseriformes, Coraciiformes, Columbiformes, and Galliformes, all of which were also considered as host groups of *P. circumflexum* based on the morphology of the observed blood stages [3]. These reports might belong to other closely related lineages of the same morphospecies. Five lineages differ from pTURDUS1 in one bp: pFALTIN14 from *Falco tinnunculus* (1) in China [11], pCIAE07 from *Circus aeruginosus* (1) in Austria, pPARUS67 from *Parus major* (1) and *Poecile palustris* (1) in Sweden [38, 55], pSFC6 from *Muscicapa striata* (1) and *Phoenicurus phoenicurus* (1) in Sweden [56, 57], and pBT7.

Most records of pBT7 (389 records in total) originate from Northern America (190) and Northern Europe (158), whereas few records stem from Eastern Asia (10), South America (9), Western Europe (9), Eastern Europe (8), Central America (2), Western Asia (1), South-Eastern Asia (1), and Southern Asia (1). Most records of pBT7 originate from passeriform birds (347 of 389 records) of the families Paridae (159), Turdidae (58), Hirundinidae (47), Corvidae (17), Muscicapidae (15), Fringillidae (16), Sylviidae (10), and Certhiidae (4). The most common host species are *Cyanistes caeruleus* (101) and *Parus major* (40), which is also the case with pTURDUS01. Lineage pBT7 was found also in Accipitriformes from Europe and Asia, in *Buteo buteo* (3), *Buteo lagopus* (1), *Buteo rufinus* (1), *Accipiter nisus* (2), *Accipiter gentilis* (1), *Accipiter gularis* (1), and *Accipiter striatus* (1) [11,14,58, present study], and in Anseriformes (19) [59] and Strigiformes (12) [34] from northern America. More than half of the pBT7 records (201/389) originate from American birds, and the host composition in the Americas differs quite strongly from that in Europe and Asia, e.g., all pBT7 records from Hirundinidae (47), Corvidae (17), Anatidae (17), Charadriiformes (2), and most records from Turdidae (45/47) and Strigidae (11/12) originate from the Americas, particularly northern America. In Central and South America, pBT7 was only found in thrushes of the genus *Catharus* spp. in their wintering habitats in Costa Rica, Belize, Colombia, and Peru [60, 61]. Apart from pTURDUS1, three other lineages are connected directly to pBT7, separated by one to three bp: pBUTBUT02 from *Accipiter nisus* (1) and *Buteo buteo* (1) in Turkey [15], pGLAHIR03 from the rufous-breasted hermit *Glaucis hirsutus* (1) in Brazil [62], and pANSANS02 from the greylag goose *Anser anser* (2) in Turkey [63].

#### *Plasmodium* clade 2: *Plasmodium* spp.

**Fig. 7:**
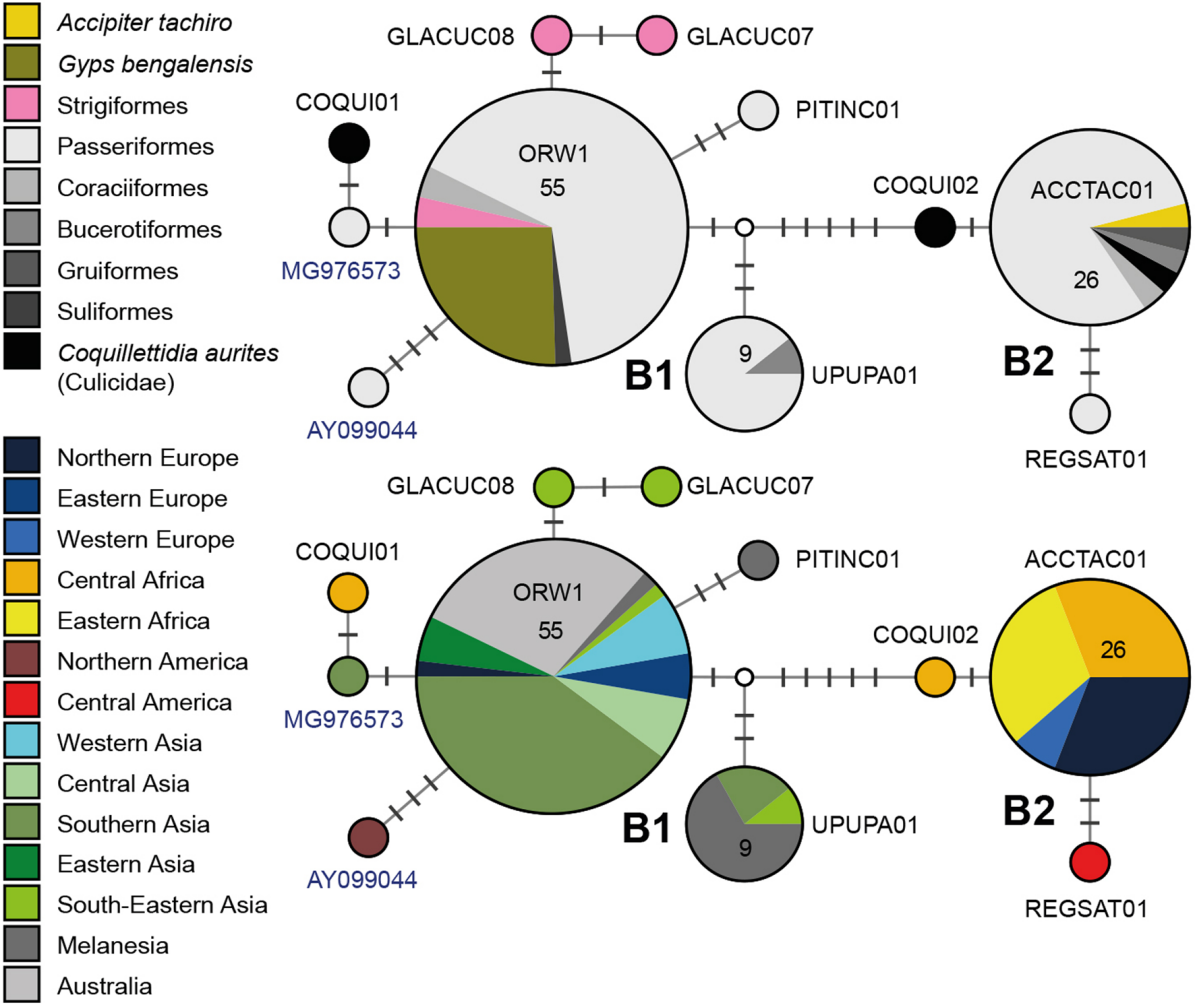
Median-Joining DNA haplotype network of partial (474 bp) *CytB* sequences belonging to a *Plasmodium* clade, whose lineages have not been studied morphologically yet. The clade features the lineages pORW1 and pACCTAC01, which were found in the white-rumped vulture *Gyps bengalis* and the African goshawk *Accipiter tachiro*, respectively. The upper image indicates the number and frequency of host species, the lower one the geographic origin according to the United Nations geoscheme. In case lineages were not listed in the MalAvi database, the GenBank accession numbers are indicated in blue

This *Plasmodium* clade features 11 lineages, two of which (pORW1 and pACCTAC01) were found in accipitriform raptors (Fig. 7). Lineage pORW1 was found in passeriform birds in Australia (14), India (7), Kyrgyzstan (4), Turkey (3), Russia (3), Armenia (1), the UK (1), Japan (1), and Myanmar (1), in coraciiform birds in Australia (2), in strigiform birds in China (2), Thailand (1), and Japan (1), and in the white-backed vulture *Gyps bengalensis* (14) in India (Additional file 3: Table S1). Poharkar et al*.* [13] detected pORW1 in 12 dead and two living individuals of *Gyps bengalensis* in the Gadchiroli district in Maharashtra (central India). Lineage pACCTAC01 was found in Passeriformes (mainly Muscicapidae) from Sweden (8), Gabon (5), Tanzania (4), and Malawi (3), and France (1), in the corncrake *Crex crex* (1) from France, in the rosy bee-eater *Merops malimbicus* (1) from Gabon, in the crowned hornbill *Tockus alboterminatus* (1) from Malawi, in the mosquito *Coquillettidia aurites* (2) from Cameroon (Additional file 3: Table S1), and in one individual of the African goshawk *Accipiter tachiro* from Gabon [64]. In Europe, pACCTAC01 was found in the passeriform species *Ficedula albicollis*, *Ficedula hypoleuca*, and *Hirundo rustica,* and in the gruiform corncrake *Crex crex*, which all migrate to wintering sites in the latter region.

#### *Plasmodium* clade 3: Plasmodium spp.

**Fig. 8:**
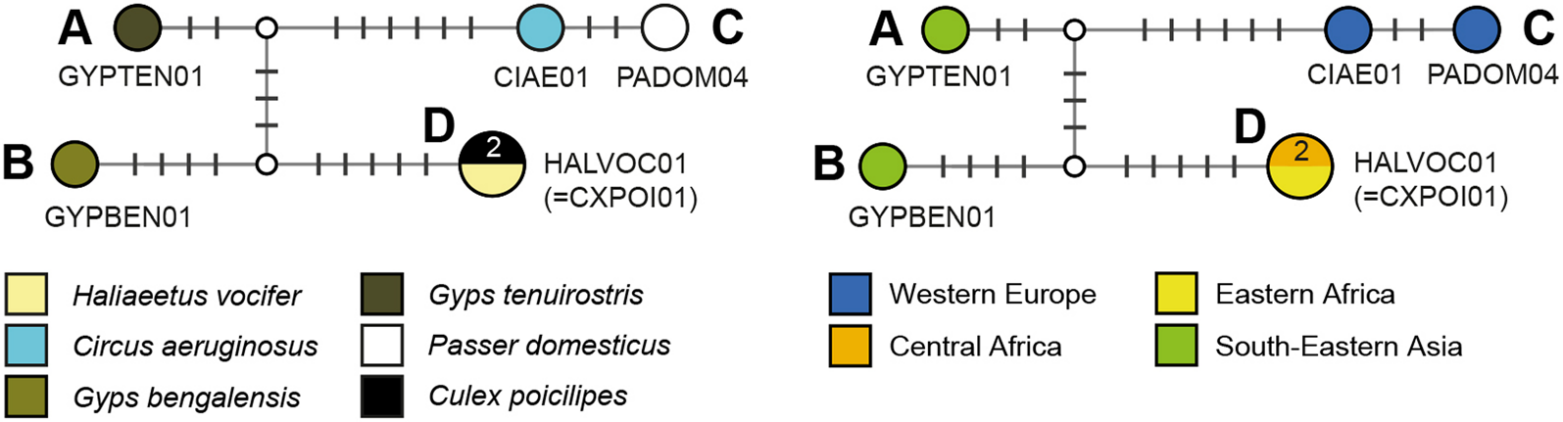
Median-Joining DNA haplotype network of partial (462 bp) *CytB* sequences belonging to a *Plasmodium* clade featuring four lineages from accipitriform raptors. None of the lineages has been characterized morphologically yet. The left image indicates the number and frequency of host species, the right one the geographic origin according to the United Nations geoscheme

This clade features five lineages in four sub-clades, which mostly originate from single species of accipitriform raptors (Fig. 8). The lineages pGYPTEN01 (sub-clade A; GenBank accession no. DQ212194) from *Gyps tenuirostris* (1) and pGYPBEN01 (sub-clade B; DQ212195) from *Gyps bengalis* (1) were found in Cambodia by Duval et al. (unpublished). The lineages pCIAE01 and pPADOM04 (sub-clade C) were found in *Circus aeruginosus* (1) from Germany [14] and *Passer domesticus* from France [65]. Last, the lineage pHALVOC01 (= CXPOI01; subclade D) was found in *Culex poicilipes* (1) from Cameroon [66] and *Haliaeetus vocifer* (1) from Uganda [67]. The alignment for this clade was trimmed to 462 bp because pGYPTEN01, pGYPBEN01, and pHALVOC01 did not cover the entire DNA barcode sequence.

#### Other *Plasmodium* lineages in accipitriform raptors

Another 17 *Plasmodium* lineages were found in one or two accipitriform birds. In nine cases, the raptors were kept in zoos or rehabilitation centres together with birds of other orders featuring these parasite lineages. Hence, accipitriform raptors are probably not natural hosts of some of these lineages.

Lineage pACCBAD01 was detected in *Accipiter badius* (2) [68] and the owls *Athene brama* (2), *Bubo sumatranus* (2) and *Tyto alba* (4) in Thailand [69]. It differs by six bp from pPESA01, which was found in Anseriformes in the USA (8), Brazil (3), and Canada (2), in the white-tipped dove *Leptotila verreauxi* (2) in Uruguay, in the passeriform *Phaeomyias murina* (1) and *Piprites chloris* (1) in Brazil, in the pectoral sandpiper *Calidris melanotos* (1) in Alaska, and one great black hawk *Buteogallus urubitinga* in Brazil [70]. *Cygnus atratus* and *Cygnus melancoryphus* infected with pPESA01 were kept together with great black hawks in the São Paulo Zoo, Brazil, and might be the source of infection [70].

Lineage pACCNIS05 was only found in *Accipiter nisus* (1) at the Beijing Raptor Rescue Centre in China [11]. It differs by one bp from *Plasmodium gallinaceum* pGALLUS01, a common parasite of the domestic chicken in Southeast Asia and one of the most thoroughly studied parasites in early haemosporidian research [3]. Although natural transmission of *P. gallinaceum* previously has not been reported from the Americas [3], lineage pGALLUS01 was also found in Tyrannidae (3) from Brazil [71] and in the common grackle *Quiscalus quiscula* (1) in the USA [72]. Recently, lineage pDENFUL02 was found in *Harpagus diodon* (1) in Brazil [73], but the sequence (MT919269) covers only 413 bp of the *CytB* barcode section in which it is identical with pACCNIS05 and pGALLUS01.

Lineage pEMSPO06 was found in *Accipiter nisus* (1) and *Otus scops* (1) at the Beijing Raptor Rescue Centre in China [11], the mallard *Anas platyrhynchos* (1) in Japan [74], the black-faced bunting *Emberiza spodocephala* (1) in South Korea [75], and the common stonechat *Saxicola torquatus* (1) in China [76]. The lineage differs by one bp from pSW2, which was linked to *Plasmodium homonucleophilum* by [77].

Lineage pMILANS05 was found in *Circus aeruginosus* (1) in Austria and in *Milvus migrans* (2) in Spain [8]. It was also found in the ruddy quail-dove *Geotrygon montana* (1) in Jamaica [78], the cattle egret *Bubulcus ibis* (1) in Africa [42], the ruff *Philomachus pugnax* in Malawi (1) and The Netherlands (1) [79], and *Culex neavei* (1) in Cameroon [66].

Lineage pPLACAS02 was found in *Accipiter tachiro* (1) and the chestnut wattle-eye *Platysteira castanea* (1) in Gabon [64], the olive sunbird *Cyanomitra olivacea* (3) in Cameroon [80, 81], and the Príncipe seedeater *Crithagra rufobrunnea* (1) and the Newton's sunbird *Anabathmis newtonii* (1) in Sao Tome and Principe [82]. Both pMILANS05 and pPLACAS02 differ by one bp from *P. elongatum* pGRW06, like several other lineages, which have not been studied morphologically yet but probably belong to *P. elongatum*. Lineage pGRW06 was found in *Buteo buteo* (1) from Austria and *Rostrhamus sociabilis* (1) from Brazil [73], whereby the sequence of the latter record (MT919272) covers only a 413 bp section of the *CytB* barcode section in which it is identical with pTRMUS02, a lineage found in one columbiform and four passeriform bird species in Brazil [62, 83, 84].

Lineage pMILANS06 was detected in *Milvus migrans* (1) in Spain [8], the Asian barred owlet *Glaucidium cuculoides* (1) in Thailand [69], the European turtle dove *Streptopelia turtur* (1) in Greece [85], the blue-spotted wood dove *Turtur afer* (1) in Gabon [82], and the European bee-eater *Merops apiaster* in Germany (2) and Portugal (1) [86]. The lineage is most similar to pNYCNYC01 (9 bp difference), which was found in the king vulture *Sarcoramphus papa* (2) and other birds in the Sao Paolo Zoo in Brazil [87]. Lineage pNYCNYC01 was also found in more than 60 birds (mainly Anseriformes) in the Americas [59, 87]. Accipitriform birds are probably not the natural hosts of these two lineages.

Lineage pMYCAME02 was recently linked to the morphospecies *Plasmodium paranucleophilum* by [88]. Tostes et al*.* [88] detected the lineage in seven avian raptor species caught in Rio de Janeiro state, southeastern Brazil, and held in captivity at the Instituto Brasileiro do Meio Ambiente e dos Recursos Naturais Renováveis (IBAMA): the accipitriform *Buteo brachyurus* (1) and *Rupornis magnirostris* (1), the strigiform *Asio clamator* (3) and *Pulsatrix koeniswaldiana* (1), and the falconiform *Falco peregrinus* (1) and *Caracara plancus* (2). Other studies recorded pMYCAME02 in the wood stork *Mycteria americana* (14) in the USA [89], in the blue-winged teal *Anas discors* (7) in the USA [59], and a few birds of other orders in South America. Lineage pMYCAME02 is part of a clade featuring numerous similar lineages, which were mainly found in the cattle egret *Bubulcus ibis* in western Africa and Southern Africa [42] and other Ciconiiformes in northern America [89]. Based on the diversity of lineages and infected bird species, the natural hosts of pMYCAME02 and related lineages are probably ciconiiform birds. The cattle egret colonized the Americas only recently in the late nineteenth century [90] and might have naturally introduced these parasites. Despite the morphological similarities, it is possible that pMYCAME02 does not belong to *P. paranucleophilum*. The parasite was originally described from one individual of *Tachyphonus* sp. (Thraupidae, Passeriformes) imported to the USA from Brazil [91], but lineage pMYCAME02 has never been detected in birds of this family. The avian raptors found infected at the IBAMA in Brazil also might not be natural hosts of this lineage.

Lineage pRHYSIM01 was found in the Blyth's hawk-eagle *Nisaetus alboniger* (1) in Thailand [92]. Moreover, it was also found in the greyish mourner *Rhytipterna simplex* (1) in Brazil [93], the yellow warbler *Setophaga petechia* in the USA [94], and the green honeycreeper *Chlorophanes spiza* (1) in Peru [93]. Similar lineages clustering with pRHYSIM01 were found exclusively in passeriform birds in the Americas.

Lineage pRTSR1 was detected in the Wahlberg's eagle *Hieraaetus wahlbergi* (1) in Malawi [95], *Milvus migrans* (1) in Spain [8], and *Falco naumanni* (20) in Spain [96]. More than 50 other records of pRTSR1 originate from passeriform birds, mainly Sylviidae and Muscicapidae in Europe and Africa.

Lineage pRUMAG01 was found in *Rupornis magnirostris* (1) in Brazil [73], but the sequence (MT919274) covers only 383 bp of the *CytB* barcode section. With 97% identity, the sequence is most similar to pPESA01, which was found in anseriform birds in the Americas [59, 70].

Lineage pSYBOR10 was found in *Circus aeruginosus* (1) in Austria, the corncrake *Crex crex* in Poland (1) and Czechia (1) [97], and passeriform birds in Sweden (12), Hungary (2), Turkey (1), Nigeria (2), Botswana (1), and Japan (1) [e.g., 98, 99]. It differs by six bp from pRTSR1.

Lineage pZEMAC01 was detected in *Aquila chrysaetos* (2) kept in the Southwest Zoo in Arizona, USA [100]. It was also found in the mourning dove *Zenaida macroura* (3) [72, 101] and the great roadrunner *Geococcyx californianus* (2) [100] in the USA, the Neotropic cormorant *Phalacrocorax brasilianus* (4) in Chile [102], and passeriform birds in Argentina (1) [62], Mexico (1) [103], the USA (1) [104], and Australia (1) [105]. The lineage differs by nine bp from *P. matutinum* pLINN1, which was also found in one individual of *Accipiter gentilis* in Austria.

Lineage pBUTBUT06 was detected in *Buteo buteo* (1) in China [11]. It resembles pTSUB01 (4 bp difference), pGALLUS02 (5 bp difference), and several other lineages, which are currently associated with *Plasmodium juxtanucleare*. The lineages in this clade were mainly detected in the domestic chicken *Gallus gallus* in Eastern Asia and Southeast Asia (Additional file 3: Table S1), but a spillover was documented to seven species of wild passeriform birds in Brazil [106].

Lineage pTEPON02 was found in the crested serpent eagle *Spilornis cheela* (1), the Asian barred owlet *Glaucidium cuculoides* (1), and the buffy fish owl *Ketupa ketupu* (1) in Thailand [68, 69], and in the common woodshrike *Tephrodornis pondicerianus* in Myanmar [75]. It is part of a clade featuring lineages detected mainly in owls in Southeast Asia.

### *Haemoproteus* parasites in accipitriform raptors

Five *Haemoproteus* species have been described from accipitriform hosts: *Haemoproteus buteonis*, *Haemoproteus catharti*, *Haemoproteus elani*, *Haemoproteus janovyi*, and *Haemoproteus nisi*. Only *H. catharti* and *H. elani* were characterized molecular genetically, but the *CytB* lineages linked to these species do not group phylogenetically into the genus *Haemoproteus*. The lineage *H. catharti* hCATAUR01 most likely belongs to the genus *Plasmodium,* while *H. elani* hBUBT1 cannot be assigned to any of the known haemosporidian genera because of its distinct sequence features. Two *Haemoproteus* clades contain lineages from accipitriform raptors, which have not been linked to morphospecies yet. The lineages in one of these clades were found exclusively in accipitriform birds, while those in the other one were mainly found in falconiform birds. *Haemoproteus buteonis* and *H. janovyi* could not be linked to any published *CytB* sequences. A summary of *Haemoproteus* lineages found in accipitriform raptors is provided in Table 6. A BI tree featuring all *Haemoproteus* lineages from accipitriform raptors is shown in Additional file 5: Fig. S4.

**Table 6:** *Haemoproteus CytB* lineages of accipitriform raptors

Lineage	Accession	Parasite species	Host species (only accipitriform birds)	Host env	Countries	References
hBUBT1	EF607291	*H. elani*	*Buteo buteo* (2), *Buteo jamaicensis* (3)	Wild	Austria, Germany, USA	[14, 112]; present study
hCIAE08	OL598532	*H.* aff. *elani*	*Circus aeruginosus* (1)	Wild	Austria	present study
hCATAUR01	MF953291	*H. catharti*	*Cathartes aura* (17)	Wild	USA	[114]
hLK03	EF564176	*H. brachiatus*	*Circus aeruginosus* (1)	Wild	Austria	present study
hBUTHEM01	MT281482	*H.* sp.	*Buteo hemilasius* (1)	Captive	China	[11]
hACCGUL01	MT281470	*H.* sp.	*Accipiter gularis* (1)	Captive	China	[11]
hACCGUL02	MT281490	*H.* sp.	*Accipiter gularis* (1)	Captive	China	[11]
hACCNIS01	MT281461	*H.* sp.	*Accipiter nisus* (1)	Captive	China	[11]
hACCNIS02	MT281462	*H.* sp.	*Accipiter nisus* (1)	Captive	China	[11]
hACCNIS03	MT281483	*H.* sp.	*Accipiter nisus* (1)	Captive	China	[11]
hBUTIND01	MT281473	*H.* sp.	*Butastur indicus* (1)	Captive	China	[11]
hBUTBUT04	MT281481	*H.* sp.	*Buteo buteo* (1)	Captive	China	[11]
hBUBIBI01	KC994901	H. obainae*	*Buteo buteo* (2)*, Accipiter nisus* (1)*, Butastur indicus* (1)	Captive	China	[11]
hMILANS01	JN164710	*H.* sp.	*Milvus migrans* (3)	Wild	Spain	[8]
hMILANS02	JN164711	*H.* sp.	*Milvus migrans* (1)*, Buteo buteo* (1)*, Buteo rufinus* (1)*, Clanga pomarina* (1)	Wild	Spain, Turkey	[8]; Yilmaz et al*.* unpub.; Simsek et al. unpub
hMILANS03	JN164712	*H.* sp.	*Milvus migrans* (6)	Wild	Spain	[8]
hAFR048	KM056451	*H.* sp.	*Milvus migrans* (1)	Wild	Malawi	[95]
unknown	MG428418	*H.* sp.	*Buteo rufinus* (1)	Unknown	Iran	Norouzian et al*.* unpub
hSTAL4	JQ768232	*H.* sp.	*Buteo buteo* (1)	Unknown	Turkey	Simsek et al*.* unpub
hCIRCUM01	KC994896	*H. noctuae*	*Buteo buteo* (1)	Captive	Austria	present study
hOTUSCO01	MT281465	*H.* sp.	*Accipiter gularis* (1)*, Circus cyaneus* (1)	Captive	China	[11]

The list features records of all *Haemoproteus CytB* lineages found in accipitriform birds, other host species are not listed. The numbers in the brackets indicate the number of individuals that featured the respective lineages. One GenBank accession number is indicated for each MalAvi lineage. The term ‘aff.’ means ‘species affinis’, indicating that the lineage is similar to other lineages, which were already linked to morphospecies. *Haemoproteus obainae might be a species inquirenda

#### *Haemoproteus* clade 1: *Haemoproteus* spp.

**Fig. 9:**
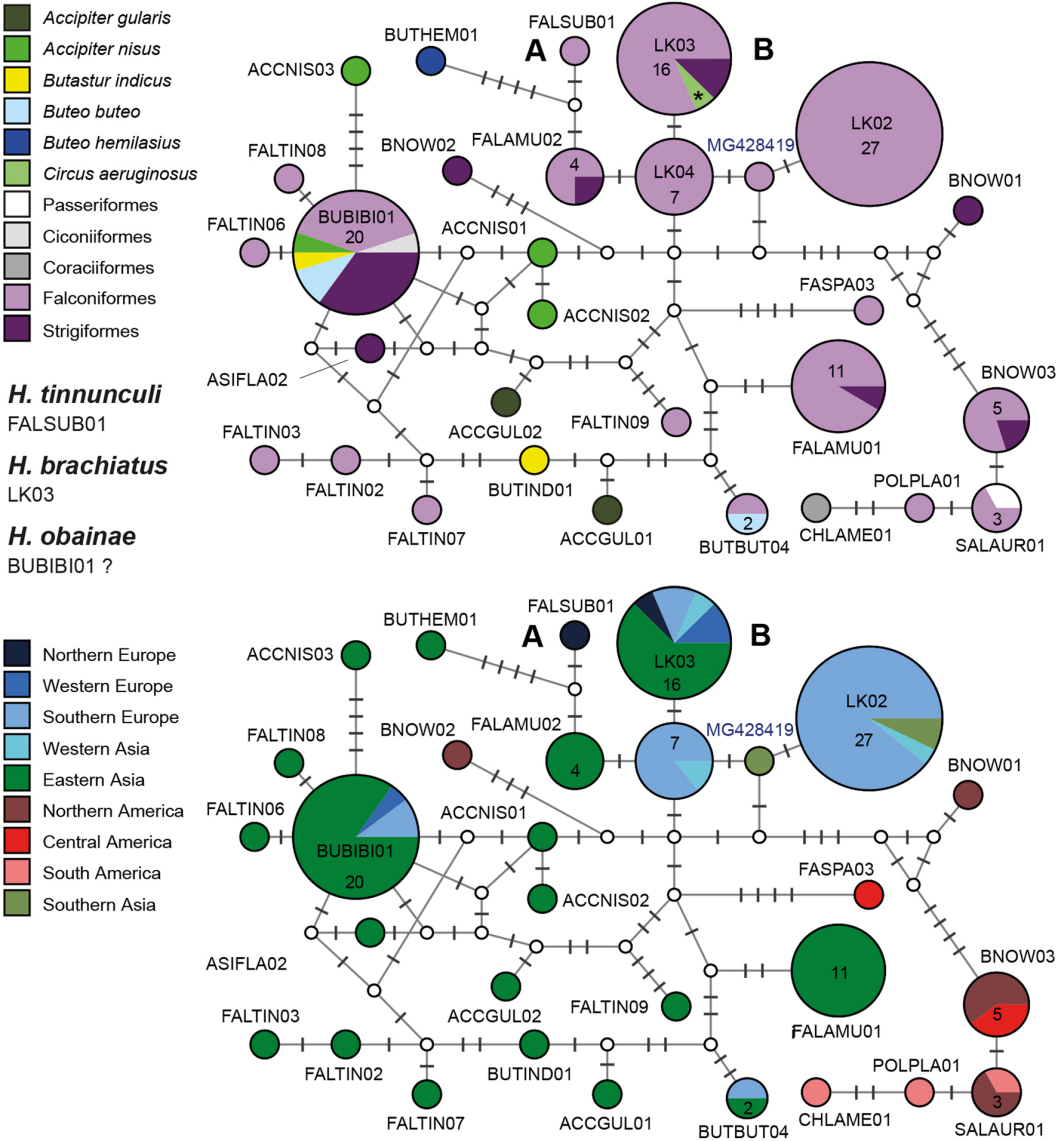
Median-Joining DNA haplotype network of partial (474 bp) *CytB* sequences belonging to the *Haemoproteus* clade featuring *Haemoproteus tinnunculi* hFALSUB01 (**A**), *Haemoproteus brachiatus* hLK3 (**B**), and several other lineages detected in Falconiformes, Strigiformes, and Accipitriformes. The upper image indicates the number and frequency of host species, the lower one the geographic origin according to the United Nations geoscheme. Asterisks mark haplotypes detected in the present study

This clade features about 30 haplotypes, which were mainly found in falconiform, strigiform, and accipitriform birds (Fig. 9). Of these, only *Haemoproteus brachiatus* hLK03 and *Haemoproteus tinnunculi* hFALSUB01 were linked to morphospecies. The latter two species were reported to be morphologically similar to *H. nisi*, which has not been characterized molecular genetically yet. *Haemoproteus brachiatus* hLK03 was found in *Falco tinnunculus* from China (11), Germany (1), and Turkey (1), in *Falco columbarius* (1) from Italy, in *Falco naumanni* (1) from Spain, in *Ninox scutulata* (1) and *Otus scops* (1) from China, and in *Circus aeruginosus* (1) from Austria. *Haemoproteus tinnunculi* hFALSUB01 is known from one specimen of the type host *Falco subbuteo* sampled in Lithuania [107]. The network contains seven lineages, which were exclusively reported from single accipitriform raptors in China by [11]: hBUTHEM01 from *Buteo hemilasius*, hACCGUL01 and hACCGUL02 from *Accipiter gularis*, hACCNIS01, hACCNIS02, hACCNIS03 from *Accipiter nisus*, and hBUTIND01 from *Butastur indicus*. Lineage hBUTBUT04 was found in *Buteo buteo* from China [11] and *Falco eleonorae* from Spain [108]. Last, lineage hBUBIBI01 was found in *Buteo buteo* (2), *Accipiter nisus* (1), and *Buteo indicus* (1), and Strigiformes (7) in China and Falconiformes in China (6), France (1), and Spain (1) [11, 109, 110]. Puech et al*.* [109] analyzed a blood sample of *Falco subbuteo* from France featuring a mixed infection with *H. brachiatus* and a new species, *Haemoproteus obainae*, but they only retrieved a sequence of lineage hBUBIBI01 by PCR and sequencing and could not assign it to either one of the two species.

#### *Haemoproteus* clade 2: *Haemoproteus* sp.

**Fig. 10:**
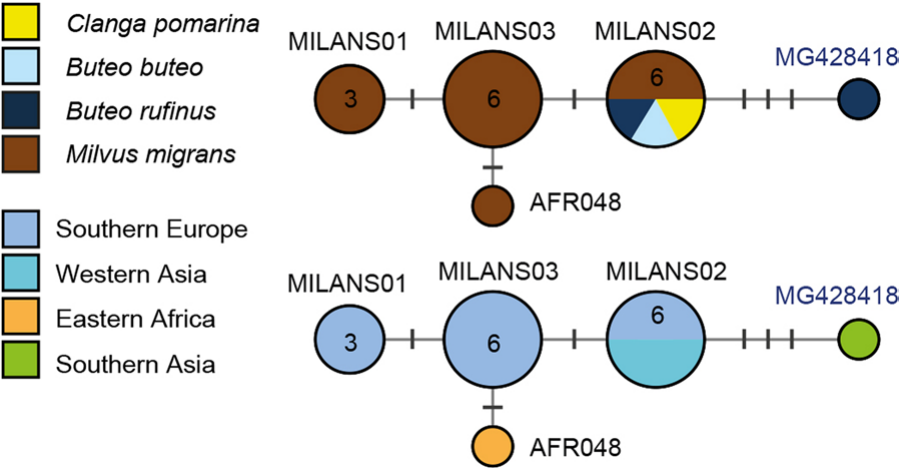
Median-Joining DNA haplotype network of partial (474 bp) *CytB* sequences belonging to a *Haemoproteus* clade featuring five lineages detected in accipitriform raptors. The upper image indicates the number and frequency of host species, the lower one the geographic origin according to the United Nations geoscheme

This clade (Fig. 10) features five similar lineages, which were exclusively reported from accipitriform raptors. The lineages hMILANS01 (3) and hMILANS03 (6) were found in *Milvus migrans* from Spain [8], hAFR048 in *Milvus migrans* (1) from Malawi [95], and pMILANS02 in *Milvus migrans* (3) from Spain [8] and *Buteo rufinus* (1, GenBank accession no. MN369025, Yilmaz et al. unpublished), *Buteo buteo* (1, MF928783, Simsek et al. unpublished), and *Clanga pomarina* (1, MH921565, Yilmaz et al. unpublished) from Turkey. A yet unnamed lineage (MG428418, Norouzian et al. unpublished) was found in one individual of *Buteo buteo* from Iran.

#### *Haemoproteus* clade 3: *Haemoproteus elani*

**Fig. 11:**
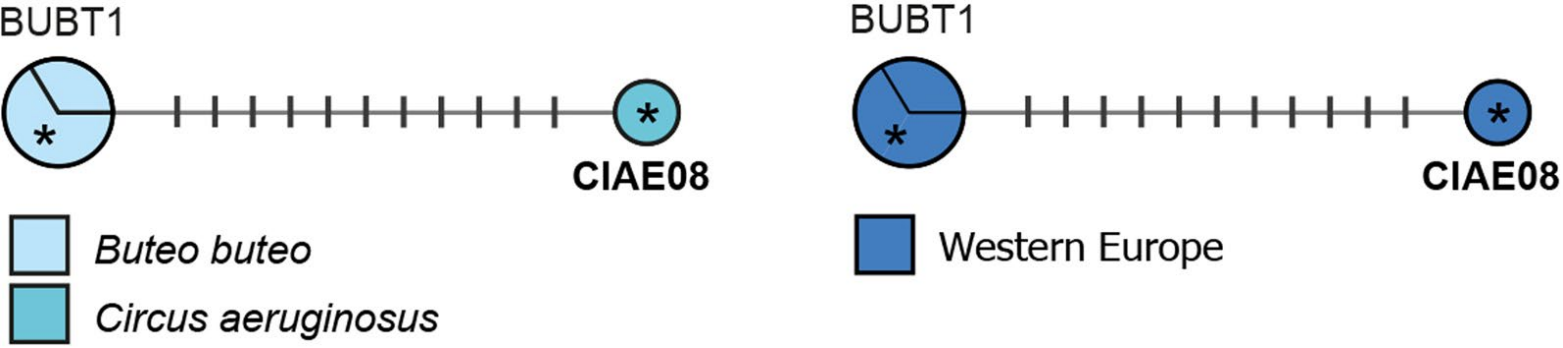
Median-Joining DNA haplotype network of partial (474 bp) *CytB* sequences belonging to a *Haemoproteus* clade featuring lineage hBUBT1, currently linked to *Haemoproteus elani.* The left image indicates the number and frequency of host species, the right one the geographic origin according to the United Nations geoscheme. Asterisks mark haplotypes detected in the present study. New lineage names are indicated in bold letters. The original hBUBT1 sequences (FJ966924, FJ966926, FJ966927) and two similar but yet unnamed lineages (FJ966920, FJ966921) from six individuals of *Buteo jamaicensis* are not included in the network because they covered only 242 bp of the *CytB* barcode region. In the 242 bp section, FJ966921 is identical to hCIAE08 and FJ966920 differs in seven bp from hBUBT1

*Haemoproteus elani* was originally described from the black-winged kite *Elanus caeruleus* (Accipitriformes) in Daman, western India by de Mello (1935). The first molecular genetic record originates from [112], who identified lineage hBUBT1 from *Buteo jamaicensis* as *H. elani*. Ishak et al*.* [112] detected three similar and yet unnamed lineages diverged by 4% from hBUBT1 in *Accipiter cooperii* (FJ966920, FJ966921, FJ966923), but the quality of blood films did not allow morphological identification. The PCR assay used in the latter study covered only 243 nucleotide sites of the standard DNA barcode region, therefore, the sequences were not included in the DNA haplotype networks (Fig. 11). Elsewhere, hBUBT1 was only found in *Buteo buteo* in Austria (2) and Germany (1) [14], and the new lineage hCIAE08 was found in *Circus aeruginosus* in Austria (present study).

#### *Haemoproteus* clade 4: *Haemoproteus catharti*

**Fig. 12:**
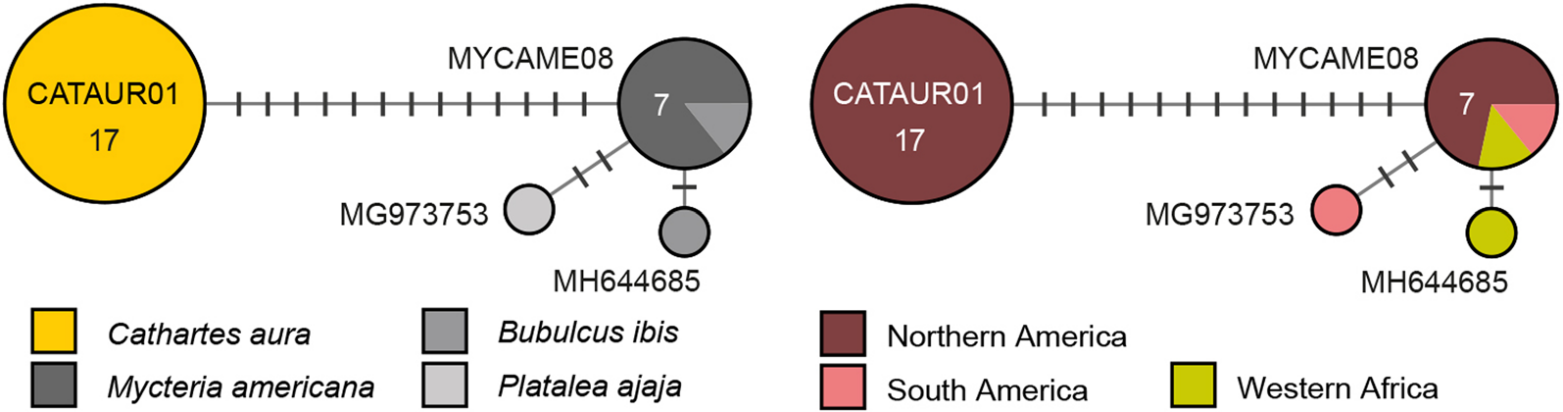
Median-Joining DNA haplotype network of partial (474 bp) *CytB* sequences belonging to a *Haemoproteus* clade featuring lineage hCATAUR01 linked to *Haemoproteus cathartic*. The left image indicates the number and frequency of host species, the right one the geographic origin according to the United Nations geoscheme

*Haemoproteus catharti* was described from the New World vulture *Cathartes aura* in South Carolina (USA) by [113]. Morphologically, mature gametocytes of *H. catharti* have some similarities with *H. tinnunculi* and *H. elani* [113, 114]. Yabsley et al*.* [114] screened blood samples of *Cathartes aura* in the USA and found 24% of 162 individuals positive for *H. catharti*. They sequenced the PCR products of 18 samples and found 17 individuals infected with hCATAUR01 and one sample with hCATAUR02 (one bp difference from hCATAUR01). Lineage hCATAUR02 covers only 367 bp of the standard *CytB* barcode region and therefore is not included in the network (Fig. 12). The two lineages are most similar to pMYCAME08 from American and African Ciconiiformes with 97% sequence similarity. The latter lineage was found in the wood stork *Mycteria americana* in the USA (5) [89] and Brazil (1) [62], and in the western cattle egret *Bubulcus ibis* (number not indicated in publication) in western Africa [42]. Two yet unnamed lineages, GenBank accession numbers MH644685 from *Buteo ibis* in western Africa [42] and MG973753 from the roseate spoonbill *Platalea ajaja* in Brazil [115], differ from hMYCAME08 by one and two bp, respectively.

#### Other *Haemoproteus* lineages in accipitriform raptors

Lineage hSTAL4 was reported from *Buteo buteo* (1) and the tawny owl *Strix aluco* (4) in Turkey by [116] and Simsek et al*.* (unpublished; MF928779-82). The present study found hCIRCUM01 in one *Buteo buteo* treated at the service unit for birds and reptiles of the Vetmeduni in Vienna. The latter lineage was found in strigiform birds in China (4) [11] and western Russia (2) [117], in the biting midges *Culicoides circumscriptus* (2) and *Culicoides submaritimus* (1) from Turkey [118], and in *Culicoides circumscriptus* from Spain (1) [119]. Lineage hOTUSCO01 was found in *Accipiter gularis* (1), *Circus cyaneus* (1), and strigiform (6) and falconiform (3) birds in the Beijing Raptor Rescue Centre in China [11].

## Discussion

This study aimed to summarize the *CytB* sequence data of haemosporidian lineages in accipitriform raptors and to show the patterns of geographic and host distribution of the parasites. This approach was also used to identify lineages, which have not been linked to morphospecies yet. The mitochondrial genomes of avian haemosporidians are highly conserved and some morphologically distinct species differ only in one or a few bp from each other in the *CytB* barcode section [21, 120]. This is particularly problematic in parasites belonging to the genus *Leucocytozoon* because the number of morphological characters accessible for species description is limited, but the genetic diversity of these pathogens is comparable to the diversity of the genus *Plasmodium* (MalAvi database; http://130.235.244.92/Malavi/). The blood stages (gametocytes) of the majority of described *Leucocytozoon* spp. parasitizing accipitriform birds are similar-sized roundish or oval bodies developing mainly in fusiform host cells with similar shape of nuclei, which is an important diagnostic character in *Leucocytozoon* spp. [3]. Gametocytes of *Haemoproteus* species and patterns of their development are also often similar in many species parasitizing accipitriform raptors. *Plasmodium* infections are common in birds belonging to the Accipitriformes birds but are often present in co-infections with *Haemoproteus* and *Leucocytozoon* spp. [3, 14]. Co-infections with haemosporidian parasites of the same and different genera are common and constitute a prominent obstacle in linking morphological data to sequence information [107, 121]. Another problem is that some groups of haemosporidian parasites specific to accipitriform raptors are not targeted by the standard primers of [6], which is the case for species of the *L. toddi* group [23] and probably most *Haemoproteus* parasites (G. Valkiūnas, pers. obs.). Specific primers have been developed already for the first group [23], but not for *Haemoproteus* lineages similar to *Haemoproteus elani* hBUBT1. The *CytB* sequence of the latter shares less than 87% identity with the *CytB* of other haemosporidians and might even be considered part of a separate subgenus or genus. These detection obstacles might explain the low prevalence of *Haemoproteus* lineages recorded in accipitriform birds so far compared to the other genera and should be considered in future studies screening this host group. Furthermore, morphological and molecular work on parasites found in accipitriform raptors has rarely been done in parallel, calling for a more integrative approach combining these techniques in future works. Therefore, at present, the available data should be treated with care. Accipitriformes are among the most endangered groups of birds, therefore investigations of haemosporidian infections are important regarding their health and preservation of biodiversity. This is particularly an alarming issue due to recent findings of damage caused by tissue stages of haemosporidian parasites in the internal organs of bird hosts [23, 122].

### *Leucocytozoon* parasites of accipitriform raptors

The authors identified 57 *Leucocytozoon* lineages, which mostly belong to either the *L. toddi* group (48 lineages) or the *L. californicus* group (4 lineages).

All 10 *Leucocytozoon* species described from accipitriform birds belong to the *L. toddi* group (Table 1), but currently, only *L. toddi*, *L. buteonis*, and *L. mathisi* can be distinguished based on the available morphological features and knowledge on the biology of these parasites [29]. Due to the similarity of blood stages of these parasites, [17] synonymized *L. mathisi*, *Leucocytozoon martyi*, *Leucocytozoon circaeti*, and *Leucocytozoon audieri* with *L. toddi*, [18] did so with *Leucocytozoon bacelari* and *Leucocytozoon franchini*, and [19] and [20] did so with *L. buteonis* and *Leucocytozoon muratovi*. Sacchi & Prigioni [123] speculated that *L. mathisi* and *L. franchini* might represent species distinct from *L. toddi*. According to [3], *Leucocytozoon beaurepairei* and *L. franchini* might be valid species, and other synonyms of *L. toddi* could be changed to valid names when more information on life cycles and DNA sequences is available. Validations and molecular characterisations of *L. buteonis* (a parasite of *Buteo* spp.) and *L. mathisi* (a parasite of *Accipiter* spp.) were conducted based on reported genetic differences and morphological features of their gametocytes and host cells. Particularly, fusiform processes of the infected blood cells are significantly longer in *L. buteonis* compared to *L. mathisi* [29]. However, the lineages linked to *L. mathisi* (lACCOP01, lACNI04) differ from each other by 4.6% and those of *L. buteonis* (lBUBT2, lBUTJAM10, lBUTREG01) by 1.5–3.4%, indicating that they might belong to several closely related cryptic species. Moreover, the parasite lineages found in the genera *Buteo*, *Accipiter*, and *Circus* cluster into several distinct subclades, indicating that each of the three raptor genera might host at least two different parasite species. Following the classification into sub-clades (Fig. 3), the currently known lineages of the *L. toddi* group might belong to about 20 distinct species. Considering that these lineages were obtained from 17 bird species only (less than 7% of 260 accipitriform species), the genetic diversity likely is much higher. Moreover, some molecular genetic studies including accipitriform birds did not detect parasites of the *L. toddi* species group [11, 14] because they used the standard PCR protocol by [6], which does not target the *CytB* of these parasites. Hanel et al*.* [12] also pointed out this problem and used the primers and PCR protocol of [72] to screen their samples more successfully. For the present study, the PCR protocol by [23] was used, which specifically targets the *CytB* of *L. toddi* group sequences. However, the latter primers also amplified the *CytB* of the lineages hBUBT1 and hCIAE08, which are currently attributed to *Haemoproteus*. The lineages of the *L. toddi* group were exclusively found in accipitriform raptors from Europe, Northern America, and Central Asia [8–10, 12]. There is one report of *L. toddi* from the falcon species *Milvago chimango* in southern Chile [124], but no illustrations of the parasites were provided. The parasite species might have been identified wrongly in the latter study because none of the *L. toddi* group lineages has been detected in falconiform birds so far. Still, no molecular genetic data is available on blood parasites of *Kaupifalco monogrammicus*, the type host of *L. toddi*.

Data on the exo-erythrocytic development of haemosporidian parasites in accipitriform raptors are absent. The present study features the first information about the exo-erythrocytic merogony of lineages attributed to *L. buteonis*. So far, meronts of *Leucocytozoon* spp. were only reported from Passeriformes, Anseriformes, Strigiformes, Podargiformes, and Sphenisciformes [3, 122], but not in diurnal raptors. The CISH analysis showed that both *L. mathisi* and *L. buteonis* preferably develop in renal tubular cells, which might indicate a typical pattern of exo-erythrocytic merogony in this species group. Although the number of meront infections was generally low in the investigated birds, higher parasite intensities could potentially damage kidneys, which should be taken into consideration in studies aiming at developing a treatment for accipitriform birds during leucocytozoonosis. Regarding blood stages of *Leucocytozoon* spp. detected with CISH, it was interesting to observe signals of elongate structures primarily in larger vessels of the heart and in lung capillaries. According to [3], gametocytes of *L. toddi* develop primarily in fusiform host cells but are exceptionally rarely observed in roundish host cells. Based on the shape of the CISH signals, the labelled parasite stages observed in larger vessels represent gametocytes in fusiform host cells. This is supported by the observation of gametocytes in histological preparations stained with haematoxylin–eosin (Fig. 1, c, i, l). Strikingly, numerous smaller roundish signals were also present but mainly observed in smaller capillaries. These stages might be young gametocytes. Valkiūnas [3] hypothesized, that gametocytes in roundish host cells might occur primarily during early parasitaemia. Although the phase of infection could not be determined for the birds investigated in this study, the birds could have died during early parasitaemia, which would explain the high numbers of small roundish signals as compared to elongated gametocytes in fusiform host cells. To confirm this hypothesis, experimental studies would be needed to clarify patterns of erythrocytic development in *L. toddi* group lineages.

The second *Leucocytozoon* clade containing multiple lineages from accipitriform raptors features *L. californicus* lFASPA02, which was recently described from *Falco sparverius* in California (USA) [31] and later found also in *Falco columbarius* in Italy [32]. However, the most common lineage in this clade is lCIAE02, which was detected in birds of ten different orders in Europe, Asia, and Africa. Half of the lCIAE02 records originate from accipitriform raptors of the genera *Milvus*, *Circus*, *Accipiter*, *Buteo*, and *Aquila*, and about one-third from seagulls *Larus* spp. Infections in birds of some orders might be abortive, i.e., sporozoites invade tissue cells leading to the formation of meronts but gametocytes do not develop. This is particularly probable in species of the genus *Larus* because in birds of this genus gametocytes of *Leucocytozoon* have been exceptionally rarely reported in blood films [125, 126]. Such abortive infections are of particular concern because they have been associated with extensive parasite multiplication in tissues and the formation of megalomeronts, causing severe damage to organs and eventually death [127]. In particular, CIAE02 was reported to cause death in a captive cobalt-winged parakeet *Brotogeris cyanoptera*, demonstrating its pathogenic potential [23]. The clade features three additional lineages from accipitriform raptors (lACCTRI01, lBUTBUT01, lCIAE06), which also have not been linked to morphospecies yet. Due to its wide host range and the potential to cause severe haemosporidiosis, morphological characterization and experimental infection studies on lCIAE02 are highly recommended.

Five *Leucocytozoon* lineages were reported from a few individuals of accipitriform raptors and were mostly found in birds belonging to orders other than Accipitriformes. Most birds featuring these lineages were kept in rehabilitation facilities together with owls, falcons, and other birds, which might be the natural hosts. Lineage lBT2 belongs to a common parasite in passeriform birds [38, 45, 46] and lBUTBUT05 was mainly found in blood-fed black flies feeding on domestic chickens, while the natural hosts of lineages lBUBO01 and lASOT06 are probably owls. The detection of the latter lineages in accipitriform birds might therefore represent cases of abortive infections. Only the lineage lMILVUS02, which was found in single individuals of *Milvus milvus*, *Haliaeetus albicilla*, *Buteo buteo*, and *Buteo lagopus* in Europe [8, present study] was not found in birds of other orders.

### *Plasmodium* parasites of accipitriform raptors

The diversity of *Plasmodium* lineages was lower (25 reported lineages) compared to that in *Leucocytozoon*. The most frequent lineages pTURDUS1 and pBT7 are linked to *P. circumflexum.* Apart from the two *P. circumflexum* lineages, only pORW1 was found in more than two individuals. The other lineages were each found in one or two individuals of accipitriform raptors but were mostly found in Passeriformes and birds of other orders. Most of these rare *Plasmodium* lineages were recorded in Eastern and South-Eastern Asia, which are rather poorly covered regarding molecular genetic studies on avian haemosporidians.

*Plasmodium circumflexum*, the type species of the subgenus *Giovannolaia*, was described from *Turdus pilaris* (Turdidae) in Germany. Morphologically similar parasites have since been reported from more than 100 species of passeriform birds, but occasionally also from species of Accipitriformes, Anseriformes, Columbiformes, Coraciiformes, Charadriiformes, Falconiformes, Strigiformes, and Galliformes. According to the literature, *P. circumflexum* is particularly common in the Holarctic and was rarely recorded in South America and Australia [3, 125, 126]. Palinauskas et al*.* [54] linked the lineage pTURDUS1 to *P. circumflexum* and suggested that pBT7 belongs to the same species. Experimental infection studies showed that parasitaemia developed in *Anas platyrhynchos*, *Carduelis spinus*, and *Loxia curvirostra* after inoculation with pTURDUS1 positive blood, however, this lineage has not been detected in wild birds of the latter three species [128]. The main hosts of pTURDUS1 and pBT7 are the two Paridae species *Cyanistes caeruleus* and *Parus major*, but both lineages were also found in accipitriform raptors and birds of other orders. However, neither pTURDUS1 nor pBT7 have been detected in Falconiformes, Columbiformes, and Piciformes, which were also reported as host groups of *P. circumflexum* by [3]. In galliform birds, pBT7 was detected in about 31 individuals of *Falcipennis canadensis*, *Tympanuchus phasianellus*, and *Bonasa umbellus* in Alaska, USA [129] (data not included in Fig. 6 due to short sequence lengths of 425 bp). There is a discrepancy in host and geographic distribution between the two lineages. More than half of the pBT7 records originate from birds in the Americas, while pTURDUS1 was only found in Old World birds. Moreover, all or most pBT7 records from Anseriformes, Strigiformes, Charadriiformes, and the passeriform families Hirundinidae, Corvidae, and Turdidae originate from the Americas. The lineages in the network form a subclade within a larger clade featuring more than 30 lineages, which differ in less than 3% from each other in the *CytB*. Most of the other common lineages were found predominantly in passeriform birds, e.g., pGRW09, pNILSUN01, pSYBOR02, pPOMFER01, and pRFF1. Lineage pGRW09 was also reported from the European bee-eater *Merops apiaster* in Portugal and Germany, confirming the presence of a *P. circumflexum*-related lineage in birds of the order Coraciiformes [86]. Lineage pSW5, which was also linked to *P. circumflexum* [130], was found mainly in Anseriformes and Gruiformes in the USA and Japan [59, 131, 132].

The second *Plasmodium* clade (Fig. 7) features the lineage pORW1, which was found mainly in passeriform birds, but also in the white-backed vulture *Gyps bengalensis* in India. Poharkar et al*.* [13] detected pORW1 in twelve dead and two living individuals of *Gyps bengalensis* in the Gadchiroli district in Maharashtra (central India). Blood film analysis (morphological data were not shown) and necropsy revealed the presence of erythrocytic meronts in the blood and tissue meronts in brain and liver sections, which were likely the cause of death for these birds [13]. Further investigations are necessary to characterise the morphology of the parasite and to test whether pORW1 and similar lineages are also found in other raptor species. The lineage pACCTAC01 was found in one individual of the African goshawk *Accipiter tachiro* from Gabon [64], but mainly in Passeriformes and birds of other orders. In Europe, pACCTAC01 was found in the passeriform species *Ficedula albicollis*, *Ficedula hypoleuca*, *Hirundo rustica,* and the corncrake *Crex crex* (Additional file 3: Table S1). Transmission might take place in Africa because all of the latter species migrate to wintering sites in this region.

The third clade features the lineages pGYPTEN01, pGYPBEN01, pCIAE01, and pHALVOC01, which were found in single individuals of *Gyps tenuirostris*, *Gyps bengalis*, *Circus aeruginosus*, and *Haliaeetus vocifer*, respectively. The sequences in this clade are similar to *Plasmodium parahexamerium* pALEDIA02 (96.2% identity), *Plasmodium multivacuolaris* pANLAT07 (95.4% identity), and other *Plasmodium* lineages, which are particularly common in African birds [133]. The morphology of these five parasite lineages still needs to be assessed because the studies publishing them did not include blood film analyses.

Another 17 *Plasmodium* lineages covering the entire *CytB* barcode section were found in one or two accipitriform birds. In at least nine cases, the raptors were kept in zoos or rehabilitation centres together with birds of other orders featuring these parasite lineages [e.g., 85,97]. Hence, accipitriform raptors are probably not natural hosts of at least some of these lineages.

### *Plasmodium* species not yet linked to CytB lineages

*Plasmodium fallax* was described from *Strix woodfordii nuchalis* (Strigiformes) in the Democratic Republic of the Congo (formerly Belgian Congo) and was reported also from the accipitriform *Accipiter nisu*s, *Aquila rapax*, *Aquila wahlbergi*, *Gyps africanus*, the passeriform *Emberiza tahapisi* and *Sylvia borin*, and the helmeted guineafowl *Numida meleagris*. Experimental observations indicate that species of three different Culicidae genera can support sporogony and might be involved in the transmission of this parasite: *Aedes aegypti, Aedes albopictus*, *Aedes atropalpus*, *Aedes triseriatus*, *Anopheles quadrimaculatus*, *Culex quinquefasciatus*, and *Culex tarsalis* [3, 134]. *Plasmodium fallax* was recorded primarily in the Ethiopian and Oriental zoogeographical regions and adjacent territories of the Palearctic. The species morphologically resembles *P. circumflexum*, but its meronts and gametocytes do not encircle the host cell nucleus and are markedly vacuolated, the erythrocytic merogony is not synchronized, and it does not complete sporogony in vectors of the genera *Culiseta* and *Mansonia* [3]. Based on the present data, it is not possible to assign any lineage identified in accipitriform birds to *P. fallax*. The diversity of *Plasmodium* lineages detected in strigiform birds is low, and so far, no molecular genetic records have been published from the type host *Strix woodfordii*. Studies on haemosporidian parasites of African birds are still rare, and there is particularly a lack of combined morphological and molecular genetic studies, which would allow assigning lineages to morphospecies.

*Plasmodium forresteri* was described from the barred owl *Strix varia* in Georgia (USA). This parasite species was also reported from *Buteo jamaicensis*, *Buteo lineatus*, *Buteo platypterus*, and *Haliaeetus leucocephalus* in Florida and Georgia [53]. Experimental infection studies showed that sporozoites develop in *Culex restuans* but not in *Culex nigripalpus*, *Culex erraticus*, and *C. quinquefasciatus*. The Japanese quail *Coturnix japonica* and the mallard *Anas platyrhynchos* were susceptible experimental hosts. Morphologically, the attenuated gametocytes of *P. forresteri*, which belongs to the subgenus *Novyella*, resemble those of *P. elongatum*, which belongs to the subgenus *Huffia* [53], but the erythrocytic meronts of *P. elongatum* predominantly develop in young red blood cells, which is not the case in *P. forresteri.* Only a few *Plasmodium* lineages have been found in single specimens of the type host: *Plasmodium* aff. *elongatum* pPADOM11, *Plasmodium* aff. *homopolare* pLAIRI01, *Plasmodium* aff. *cathemerium* pSEIAUR01, *Plasmodium* sp. pSTVAR04, and *P.circumflexum* pSW5 [35, 135], whereas not even a single *Plasmodium* lineage has been reported from the four additional assumed natural hosts in North America. So far, *P.* cf. *circumflexum* pBT7 is the only *Plasmodium* lineage, which has been found in several species of accipitriform and strigiform raptors in Northern America, although not in the same host species as *P. forresteri*.

Three *Plasmodium* species were described from accipitriform raptors by [136] in Israel, *P. accipiteris* and *P. alloelongatum* from adult *Accipiter brevipes*, and *P. buteonis* from a juvenile *Buteo buteo*. The morphology of *P. accipiteris* erythrocytic meronts is reminiscent of *Plasmodium tenuis* and *Plasmodium merulae*, which are both considered subspecies of *Plasmodium vaughani* [3], but the gametocytes of *P. accipiteris* possess refractive globules, which is a distinct character indicating the validity of this species. However, *P. alloelongatum* is probably a synonym of *P. elongatum,* and *P. buteonis* needs re-description [16].

### *Haemoproteus* parasites of accipitriform raptors

We identified 21 *Haemoproteus* lineages in accipitriform raptors, the majority of which was found in single individuals. Most of the lineages are part of one of the four clades shown as DNA haplotype networks.

The first *Haemoproteus* clade (Fig. 9) features 30 lineages, which were almost exclusively found in falconiform, strigiform, and accipitriform birds. Among those, only two lineages were linked to morphospecies, *Haemoproteus brachiatus* hLK03 and *Haemoproteus tinnunculi* hFALSUB01 from *Falco tinnunculus* [107]. Puech et al*.* [109] analysed a blood sample of *Falco subbuteo* from France featuring a mixed infection with *H. brachiatus* and a new species, *Haemoproteus obainae*, but they only retrieved a sequence of the lineage hBUBIBI01 by PCR and sequencing and could not assign it to either one of the two species. Based on the original description, *Haemoproteus obainae* might be a *species inquirenda* (a parasite of doubtful identity), and further data are needed to clarify its taxonomic validity. Ten of the lineages in this clade were detected in accipitriform raptors, among those eight in single individuals kept in the Beijing Raptor Rescue Centre in China [11]. Huang et al*.* [11] also prepared blood films from most of their samples, but no pictures are provided in the publication. Combined molecular genetic and morphological analyses of a broader sample of Eastern Asian raptors are required to confirm that these birds are natural hosts of these lineages. Alternatively, the source of infection could be falcons and owls kept in the same rehabilitation facility.

The second *Haemoproteus* clade (Fig. 10) features five similar lineages, which were exclusively reported from the accipitriform raptors *Milvus migrans*, *Buteo rufinus*, *Buteo buteo*, and *Clanga pomarina* (hMILANS01, pMILANS02, hMILANS03, hAFR048, and MG428418). No morphological data has been published for any of the five lineages so far. The sequences contained within this clade differ by more than 4% from other *Haemoproteus* lineages and seem to be specific to accipitriform birds. The lineages found in this clade are the only *Haemoproteus* sequences found in *Milvus migrans* and *Buteo rufinus* to date. Pérez-Rodríguez et al*.* [8] also screened more than 200 individuals of *Milvus milvus* in Spain and did not find any bird infected with *Haemoproteus* spp. In contrast to *Milvus migrans*, *Milvus milvus* is not native in sub-Saharan Africa. Since the European populations of the four raptors species hosting these parasite lineages have wintering sites in either Western or Eastern Africa, transmission probably takes place there and not in Europe. To clarify the taxonomic status of these parasites, morphological analyses of gametocytes of *Milvus milvus* are required.

*Haemoproteus elani* was described from the black-winged kite *Elanus caeruleus* (Accipitriformes) in Daman, western India, and was reported from numerous other accipitriform raptors in North America, Europe, Africa, Middle East, and Asia [3, 111, 137]. Ishak et al*.* [112] identified lineage hBUBT1 from *Buteo jamaicensis* as *H. elani*. The linkage of hBUBT1 to *Haemoproteus elani* might be incorrect, because the type host *Elanus caeruleus* belongs to a different genus and inhabits Eurasia and Africa, but not the Americas. The lineage hBUBT1 and similar sequences form a unique clade and share less than 87% identity with the *CytB* of other haemosporidians, preventing the unequivocal assignment to any of the known genera. Further analyses are required to determine the generic position and biological features of these parasites. *Haemoproteus elani* was reported to be one of the most common *Haemoproteus* parasites in accipitriform raptors, but molecular genetic studies reported lineage hBUBT1 and related ones from a few bird species only. Both ‘nest 2’ reverse primers (HaemR2 and HaemR2L) of the standard PCR protocol by [6] feature four mismatches in the 3’-region to lineages of this group, which might explain the extremely scarce molecular genetic record in other studies. The sequences of the present study were obtained using the primers by [23], which were originally designed to target lineages of the *L. toddi* species group. Sequencing larger *CytB* sections would allow identifying better primer sites for this group of haemosporidians.

The systematic position of *Haemoproteus catharti* also needs to be addressed. *Haemoproteus catharti* was described from the New World vulture *Cathartes aura* in South Carolina (USA) by [113]. Yabsley et al*.* [114] linked hCATAUR01 and hCATAUR02 to *Haemoproteus catharti*. The latter lineages are part of the *Plasmodium* clade, but the parasite identified as *Haemoproteus catharti* did not feature erythrocytic meronts and therefore was considered a member of the genus *Haemoproteus* by [114]. However, the original description of *Haemoproteus catharti* was based on blood films featuring co-infections with at least two different *Plasmodium* species [113]. Although the description of *Haemoproteus catharti* is valid, the linked lineages hCATAUR01 and hCATAUR02 likely belong to a *Plasmodium* species present in co-infection.

Three other *Haemoproteus* lineages were detected in one or two individuals, hSTAL4 in *Buteo buteo* from Turkey (Simsek et al*.*, unpublished; MF928778), hCIRCUM01 in *Buteo buteo* from Austria, and hOTUSCO01 in *Accipiter gularis* and *Circus cyaneus* from China [11]. The latter three lineages are part of a diverse *Haemoproteus* clade, which almost exclusively features lineages found in strigiform birds. All accipitriform raptors featuring these lineages were kept indoors close to owls, which presumably serve as reservoir hosts for *Culicoides* vectors. It is unclear if the parasites complete development and produce gametocytes in accipitriform birds.

### *Haemoproteus* species not yet linked to *CytB* lineages

*Haemoproteus buteonis* was described from *Buteo buteo* in Sweden. The parasite was reported from accipitriform birds in the Holarctic (mainly Palearctic), namely *Accipiter cooperii*, *Accipiter nisus*, *Aquila nipalensis*, *Buteo platypterus*, *Circus aeruginosus*, *Pernis apivorus*, and *Pernis ptilorhynchus* [3]. Peirce et al*.* [137] considered the parasite species a synonym of *H. elani*, but Valkiūnas [3] pointed out some morphological differences and suggested treating *H. buteonis* as a distinct species. Due to the morphological similarities and overlapping host ranges, *H. buteonis* might be related to *H. elani*. The parasite has not yet been characterized using molecular genetics, but *H. buteonis* might feature a lineage related to hBUBT1, which was attributed to *H. elani* by [112]. If *H. buteonis* is closely related to hBUBT1, which features unique *CytB* sequences not clustering with those of the other genera, it might not have been detected in studies using the standard primers by [6].

*Haemoproteus janovyi* was described from *Gyps africanus* in northwest Zimbabwe. The parasite is common in African vultures, and morphologically similar gametocytes were found throughout southern Africa in *Necrosyrtes monachus*, *Torgos tracheliotus*, *Trigonoceps occipitalis*. The gametocytes of *H. janovyi* resemble those of *H. nisi* and *H. tinnunculi* because they also might fully encircle the host nucleus [3, 138]. So far, *H. janovyi* neither has been characterized using molecular genetics nor were any haemosporidian *CytB* sequences published from the vulture species reported to host this parasite. Blood samples of African vultures were collected extensively in recent ornithological studies [139–141], but no parasite screenings were performed.

*Haemoproteus nisi* was described from *Accipiter nisus* in Scotland, UK, and was found in numerous accipitriform hosts in the Holarctic, Ethiopian, and Oriental zoogeographical regions (Table 1) [3]. This parasite is certainly common in *Accipiter nisus* in Eurasia [142]. Due to the presence of circumnuclear fully-grown gametocytes, *H. nisi* resembles *H. brachiatus*, *H. janovyi*, and *H. tinnunculi* [3], but a molecular characterization is still missing. The lineages linked to *H. brachiatus* hLK03 and *H. tinnunculi* hFALSUB01 are part of a clade, which features almost 30 haemosporidian lineages of falconiform, strigiform, and accipitriform raptors (Fig. 9). This clade contains all *Haemoproteus* lineages found in *Accipiter nisus* so far, hACCNIS01, hACCNIS02, hACCNIS03, and hBUBIBI01. The first three lineages were found in single individuals of *Accipiter nisus* in China [11], while hBUBIBI01 was found also in *Buteo buteo, Butastur indicus*, *Falco tinnunculus*, *Asio otus*, *Athene noctua*, and *Bubo bubo* in China [11], and in *Falco subbuteo* and *Falco eleonorae* in Europe [109, 110]. Given that hBUBIBI01 features a different host composition than proposed for *H. nisi* and that the other lineages were recorded only once in China, they are probably not associated with *H. nisi*, but might be abortive infections in this host. Blood film-positive samples of *Accipiter nisus* were negative in PCRs using the primers by [6], therefore *H. nisi* and related parasites might not be targeted by this PCR assay (G. Valkiūnas, pers. obs.).

### Taxonomic problems

Despite the relatively small number of described haemosporidian species in Accipitriformes birds, only a few of them have been characterized using molecular genetics. These species are *L. buteonis* lBUBT2 (lBUTJAM10, lBUTREG01), *L. mathisi* lACCOP01 (lACNI04), and *H. elani* hBUBT1. The only other molecular genetically characterized species reported to be common in accipitriform birds, but first described from a different host group, is *P. circumflexum* pTURDUS1 (pBT7) (see also Table 1). However, the different lineages linked to *L. buteonis* and *L. mathisi* differ strongly in the *CytB* barcode region and were found in different host species, thus indicating they might belong to several cryptic species, and both parasite names might cover species groups, as is the case with the *L. toddi* group. This needs further research. The host distribution of the lineages in the *L. toddi* species group and the genetic distance between them support the presence of about 20 parasite species in this clade. Some of the known lineages might belong to other *L. toddi* group species, which were synonymized with *L. toddi* based on the similarity of their gametocytes and host cells [3], but the original descriptions of these species are incomplete and the type material is absent for all of them [29]. Moreover, most of these species were originally described from birds in Africa, which yet have not been screened for avian haemosporidians using molecular genetics. Valkiūnas et al*.* [29] and Sehgal et al*.* [9] suggested that *L. toddi* is likely a group of cryptic species, with different species or subspecies infecting *Buteo* spp. and *Accipiter* spp. However, adding the new data published by [12] and the present study shows a more complex picture. Both *Buteo* spp. and *Accipiter* spp. feature lineages in at least three separate sub-clades and *Milvus* spp. and *Circus* spp. in at least two. Some lineages were found in birds of different genera, but most seem specific to single bird species even. Characterizing the species diversity in this parasite group based on morphology is particularly complicated due to the low number of distinctive features and overlapping ranges of their morphometric characters. Analyses of exo-erythrocytic parasites stages in bird hosts and sporogonic stages in Simuliidae vectors might reveal additional morphological features for future taxonomic studies, which could facilitate species delimitation. The problem with *H. catharti* hCATAUR01 is that the lineage clusters in the *Plasmodium* clade and probably originates from *Plasmodium* sp. present in the same samples. Another issue that needs to be addressed is that hBUBT1, the *CytB* lineage linked to *H. elani* by [112], does not cluster phylogenetically into the *Haemoproteus* clade. It shares less than 87% identity with lineages of the three main genera, not allowing a clear assignment to any known haemosporidian genus. Despite *H. elani* and the morphologically similar species *H. buteonis* having been reported frequently from accipitriform birds, the number of molecular genetic records is low. The fact that the standard DNA barcode primers by [6] generally do not allow amplification of the *CytB* in this group, might partially explain that it was rarely detected in molecular genetic parasite screenings. The development of new PCR assays specifically targeting these lineages would be highly recommended. *Plasmodium circumflexum* was also commonly reported from accipitriform raptors and the observation of gametocytes indicates that the parasite completes its life cycle in this host group (G. Valkiūnas, pers. obs.). However, the CISH conducted on tissue samples of five raptors (*Accipiter nisus*, *Buteo buteo* and *Circus aeruginosus*) infected with pTURDUS1 or pBT7 was negative and neither tissue meronts nor erythrocytic parasite stages were observed. The reason for the contrasting results between PCR and CISH remains unclear but might relate to a lower detection rate of CISH compared to sensitive PCR assays in case of low parasite intensities. Hanel et al*.* [12] detected pTURDUS1 in a single adult individual of *Accipiter gentilis* in Czechia by PCR and sequencing, but also did not observe parasites in the blood films. The latter might be cases of abortive infections in which sporozoites were present in the blood but host cells were not infected [143]. Gametocytes and erythrocytic meronts of *P. circumflexum* were observed in a wild-caught juvenile *Accipiter badius* in Thailand, however, the lineage detected was pACCBAD01, differing by 3.1% from pTURDUS1 in the *CytB* [144].

### Accidental infections and contaminations

Most of the *Leucocytozoon* lineages detected in accipitriform raptors, particularly the lineages contained in the *L. toddi* clade, are specific to this host group. On the contrary, most *Plasmodium* lineages were found mainly in birds of other host orders, e.g., pACCTAC01, pBT7, pLINN1, pPLACAS02, pTURDUS1, pACCTAC01 in Passeriformes, pNYCNYC01 and pPESA01 in Anseriformes, pRTSR1 in Falconiformes, pACCNIS05 in Galliformes, and pACCBAD01 in Strigiformes. Some *Haemoproteus* lineages were also predominantly reported from other birds, e.g., hSTAL4, hCIRCUM01, and hOTUSCO01 from Strigiformes, hBUBIBI01 from Strigiformes and Falconiformes, and hLK03 from Falconiformes. Most accipitriform raptors featuring the latter lineages were kept in raptor rescue centres, zoos, or animal hospitals together with strigiform and falconiform birds as well as birds of other orders. Dipteran vectors then might transmit parasites between birds of different orders when they are kept in close proximity. Moreover, the birds might be exposed to dipteran vectors, which they do not or rarely encounter in their natural habitats, facilitating accidental infections in untypical host species. Parasites featuring some of these lineages might not complete their life cycles in accipitriform raptors, but the sporozoites injected by blood-feeding vectors still might be detected for a certain period using sensitive PCR assays. For example, living sporozoites of *Leucocytozoon* spp. were observed in the blood of experimentally infected birds for up to 11 days post infection [143, 145]. Valkiūnas et al*.* [133] found that several yellow-whiskered greenbuls *Andropadus latirostris* from Ghana were positive for *Leucocytozoon* spp. using the nested PCR protocol by [6], however, microscopic analysis of the blood films showed only sporozoites in the blood. Valkiūnas et al*.* [143] suggested that sensitive PCR-screenings potentially also detect the presence of haemosporidian sporozoites in the hosts’ blood. Therefore, the detection of these lineages in molecular genetic parasite screenings does not necessarily prove that accipitriform raptors are susceptible hosts. Unless blood stages visualization confirms the presence of gametocytes, accipitriform raptors should be treated as provisional hosts of these lineages. Abortive development has been documented in haemosporidians. In this case, tissue stages might develop partly and produce templates for PCR amplification, but tissue merozoites either do not develop or are incapable to infect blood cells [23, 127].

Another explanation for the detection of unusual haemosporidian lineages in birds is contamination. Bensch et al*.* [146] recently discussed some cases in avian haemosporidian parasite screenings. Common causes of unusual haemosporidian reports might be contaminations of laboratory chemicals (e.g., PCR reagents and extraction buffers) with PCR products or DNA from positive controls, and the confusion of samples [146]. Some of the lineages found in accipitriform raptors, particularly those belonging to *Plasmodium* spp., might result from contaminations because they were rarely reported in raptors and mostly found in other bird groups.

## Conclusion

The present study summarizes and discusses information on *CytB* sequences of haemosporidian parasites detected in accipitriform raptors worldwide. So far, only five of the 21 haemosporidian parasites described from or reported to occur in Accipitriformes birds have been characterized molecular genetically, and there are numerous taxonomic questions, which deserve attention. The total number of *CytB* lineages recorded in accipitriform birds was 57 for *Leucocytozoon*, 25 for *Plasmodium*, and 21 for *Haemoproteus*. The DNA haplotype networks visualize the geographic and host distribution of most lineages and suggest directions for taxonomy research. They allowed identifying numerous distinct groups of lineages, which have not been linked to morphospecies yet, and many of them likely belong to yet undescribed parasite species. Whereas the majority of *Leucocytozoon* and *Haemoproteus* lineages are specific to this host group, most *Plasmodium* lineages were predominantly found in birds of other orders. Some of these lineages are common in owls and other birds, which are often kept at the same facilities as accipitriform raptors (raptor rescue centres and zoos), suggesting local transmission and probably abortive infections of accipitriform raptors with owl parasites. Several taxonomic and systematic problems could not be resolved in the present study. To clarify these issues, combined morphological and molecular genetic analyses on a wider range of accipitriform host species, including the examination of type hosts, are recommended. Some general primers commonly used in haemosporidian research do not allow the detection of many *Haemoproteus* and *Leucocytozoon* lineages specific to accipitriform hosts. This calls for the development of new PCR protocols for the detection of haemosporidians in this bird group.

## Supplementary Information

**Additional file 1**: **Fig. S1.** An intramuscular parasite cyst (arrow) detected in a hematoxylin-eosin stained section of the heart of a western marsh harrier *Circus aeruginosus *co-infected with *Leucocytozoon *sp. lCIAE03 and *P. circumflexum *pTURDUS1. The morphology resembles tissue cysts of parasites belonging to the family Sarcocystidae (Conoidasida, Apicomplexa). Scale bar is 50 μm.

**Additional file 2**: **Fig. S2.** Bayesian inference tree of *CytB *lineages (474 bp) belonging to the genus *Leucocytozoon*, except for the sequences belonging to the *Leuocytozoon toddi *species group, which are shown in Fig. 2. Bayesian posterior probabilities and Maximum likelihood bootstrap values are indicated at most nodes. A sequence of *L. buteonis *(belonging to the *L. toddi *species group) was used as outgroup. The scale bars indicate the expected number of substitutions per site according to the model of sequence evolution applied. Lineage names in bold letters were found in accipitriform raptors. Species names indicate that the lineages were already linked to the respective morphospecies.

**Additional file 3: Table S1.** *CytB* sequences used in the present study (including GenBank accession numbers, MalAvi lineage names, localities, host species, and references).

**Additional file 4**: **Fig. S3.** Bayesian inference tree of *CytB *lineages (474 bp) belonging to the genus *Plasmodium*. Bayesian posterior probabilities and Maximum likelihood bootstrap values are indicated at most nodes. A sequence of *Haemoproteus tinnunculi *was used as outggroup. The scale bars indicate the expected number of substitutions per site according to the model of sequence evolution applied. Lineage names in bold letters were found in accipitriform raptors. Species names indicate that the lineages were already linked to the respective morphospecies.

**Additional file 5**: **Fig. S4.** Bayesian inference tree of *CytB *lineages (474 bp) belonging to the genus *Haemoproteus*. Bayesian posterior probabilities and Maximum likelihood bootstrap values are indicated at most nodes. The sequences of the *H. elani *clade were used to root the tree. The scale bars indicate the expected number of substitutions per site according to the model of sequence evolution applied. Lineage names in bold letters were found in accipitriform raptors. Species names indicate that the lineages were already linked to the respective morphospecies.

## Data Availability

The dataset supporting the conclusions of this article is included within the article and its additional files.

## Abbreviations

B.-H.: Bosnia-Herzegovina
BI: Bayesian inference
bp: Base pair/s
CISH: Chromogenic in situ hybridization
*CytB*: *Cytochrome b*
ML: Maximum likelihood
rpm: Rotations per minute
