# Supplementary material for: Avian haemosporidian parasites of accipitriform raptors
Source: Malar J. 2022 Jan 5;21:14. doi: 10.1186/s12936-021-04019-z (PMC8729155; doi:10.1186/s12936-021-04019-z)
Supplement: Supplementary file 1 — Additional file 1: Fig. S1. An intramuscular parasite cyst (arrow) detected in a hematoxylin-eosin stained section of the heart of a western marsh harrier Circus aeruginosus co-infected with Leucocytozoon sp. lCIAE03 and P. circumflexum pTURDUS1. The morphology resembles tissue cysts of parasites belonging to the family Sarcocystidae (Conoidasida, Apicomplexa). Scale bar is 50 μm. [file 12936_2021_4019_MOESM1_ESM.pdf]

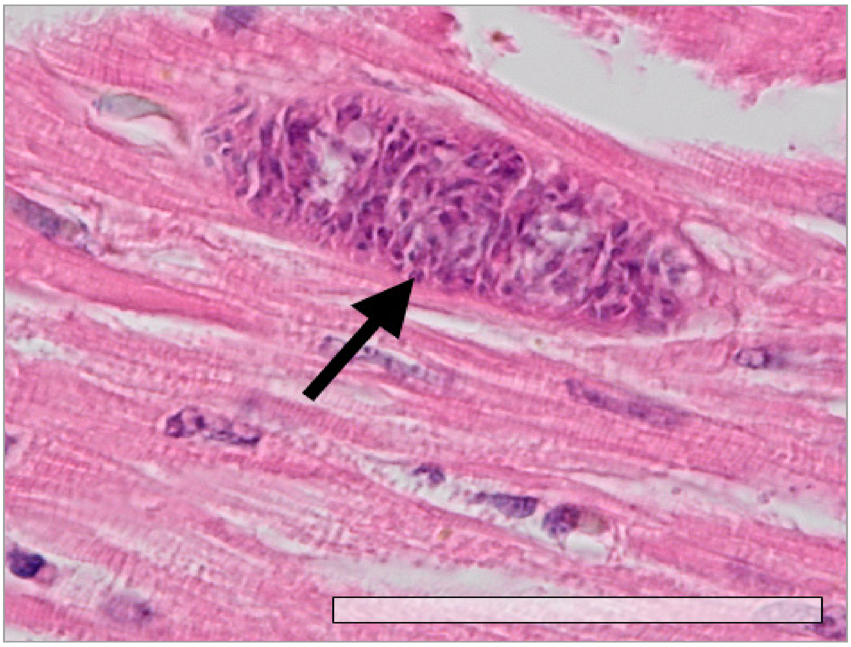

Additional file 1: Figure S1. An intramuscular parasite cyst (arrow) detected in a hematoxylin -eosin stained section of the heart of a western marsh harrier *Circus aeruginosus* co-infected with *Leucocytozoon* sp. lCIAE03 and *P. circumflexum* pTURDUS1. The morphology resembles tissue cysts of parasites belonging to the family Sarcocystidae (Conoidasida, Apicomplexa).
Scale bar is 50μm.
