# Supplementary material for: Avian haemosporidian parasites of accipitriform raptors
Source: Malar J. 2022 Jan 5;21:14. doi: 10.1186/s12936-021-04019-z (PMC8729155; doi:10.1186/s12936-021-04019-z)
Supplement: Supplementary file 2 — Additional file 2: Fig. S2. Bayesian inference tree of CytB lineages (474 bp) belonging to the genus Leucocytozoon, except for the sequences belonging to the Leuocytozoon toddi species group, which are shown in Fig. 2. Bayesian posterior probabilities and Maximum likelihood bootstrap values are indicated at most nodes. A sequence of L. buteonis (belonging to the L. toddi species group) was used as outgroup. The scale bars indicate the expected number of substitutions per site according to the model of sequence evolution applied. Lineage names in bold letters were found in accipitriform raptors. Species names indicate that the lineages were already linked to the respective morphospecies. [file 12936_2021_4019_MOESM2_ESM.pdf]

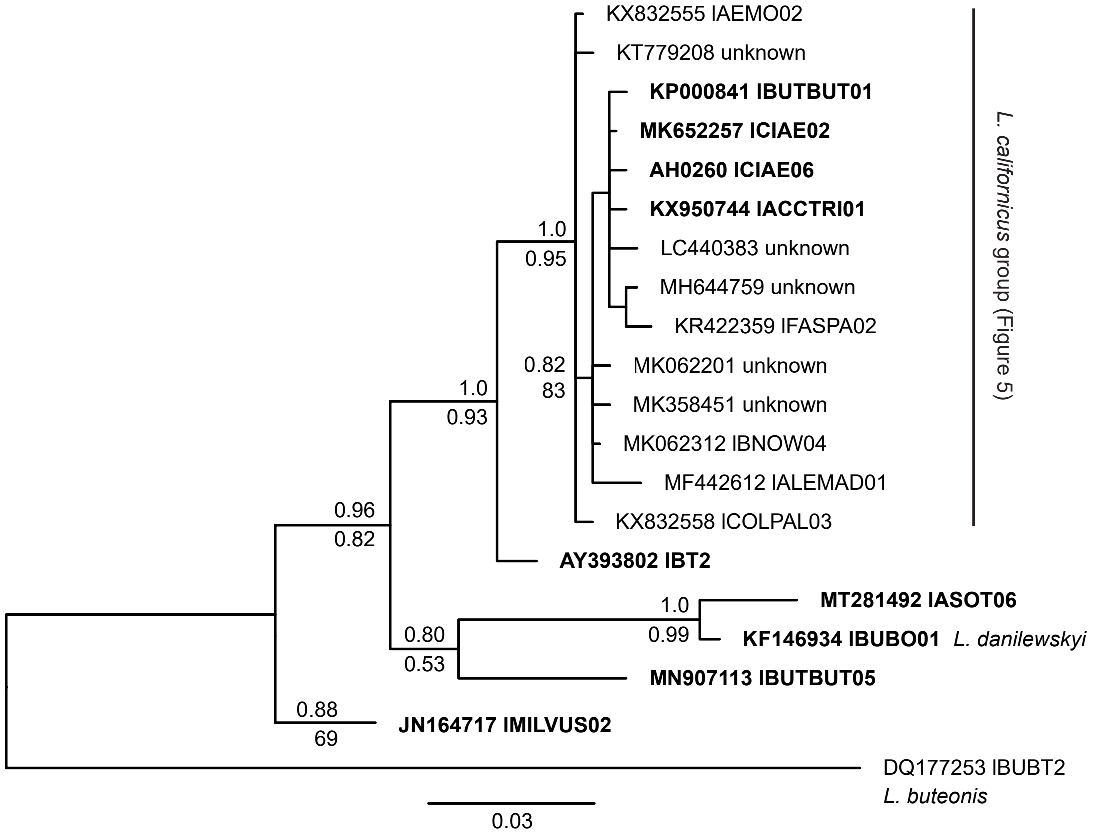

Additional file 2: Figure S2

Bayesian inference tree of *CytB* lineages (474 bp) belonging to the genus *Leucocytozoon*, except for the sequences belonging to the *Leuocytozoon toddi* species group, which are shown in Figure 2. Bayesian posterior probabilities and Maximum likelihood bootstrap values are indicated at most nodes. A sequence of *L. buteonis* (belonging to the *L. toddi* species group) was used as outgroup. The scale bars indicate the expected number of substitutions per site according to the model of sequence evolution applied. Lineage names in bold letters were found in accipitriform raptors. Species names indicate that the lineages were already linked to the respective morphospecies.
