# Supplementary material for: Avian haemosporidian parasites of accipitriform raptors
Source: Malar J. 2022 Jan 5;21:14. doi: 10.1186/s12936-021-04019-z (PMC8729155; doi:10.1186/s12936-021-04019-z)
Supplement: Supplementary file 5 — Additional file 5: Fig. S4. Bayesian inference tree of CytB lineages (474 bp) belonging to the genus Haemoproteus. Bayesian posterior probabilities and Maximum likelihood bootstrap values are indicated at most nodes. The sequences of the H. elani clade were used to root the tree. The scale bars indicate the expected number of substitutions per site according to the model of sequence evolution applied. Lineage names in bold letters were found in accipitriform raptors. Species names indicate that the lineages were already linked to the respective morphospecies. [file 12936_2021_4019_MOESM5_ESM.pdf]

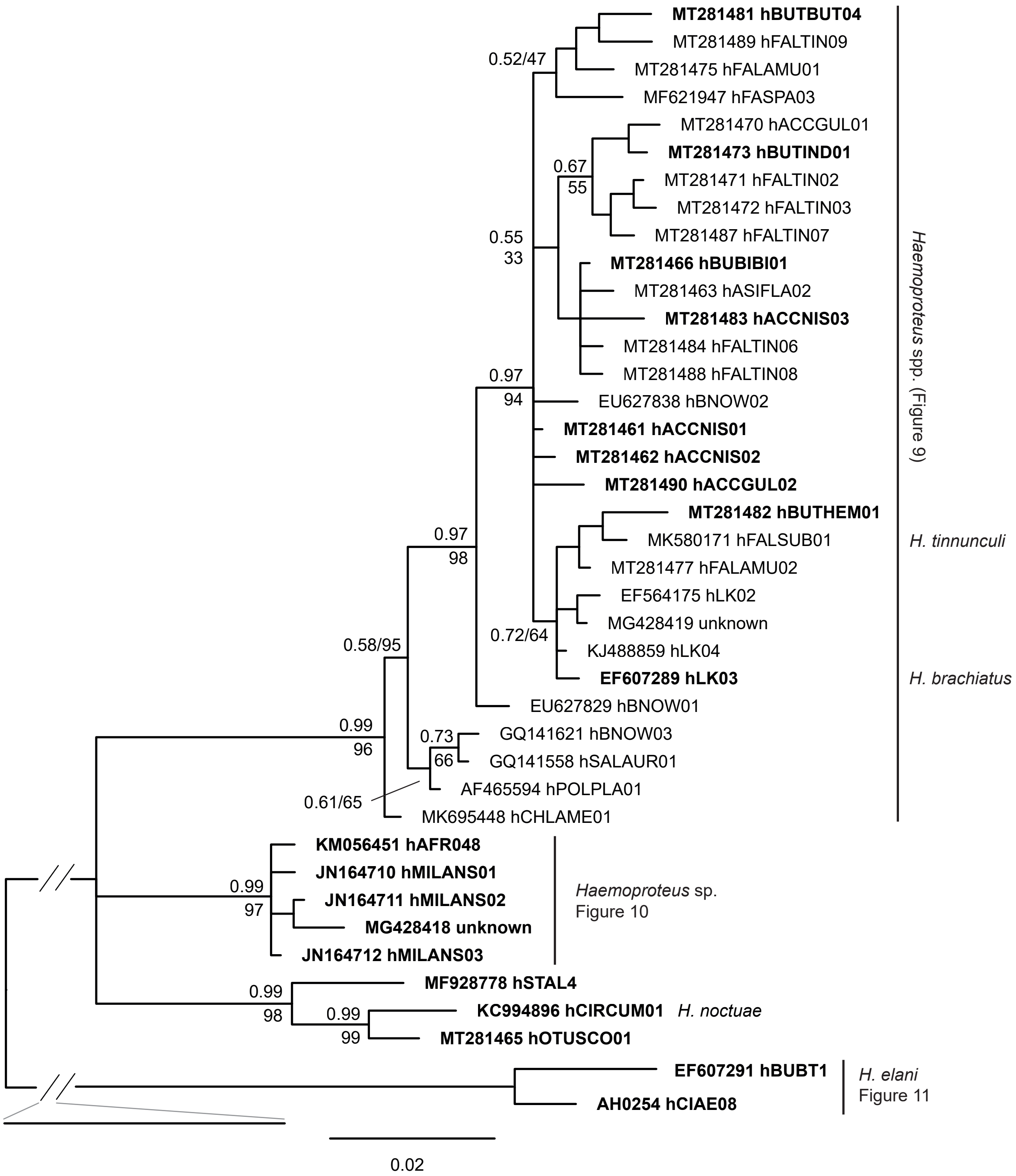

Additional file 5: Figure S5

Bayesian inference tree of *CytB* lineages (474 bp) belonging to the genus *Haemoproteus*. Bayesian posterior probabilities and Maximum likelihood bootstrap values are indicated at most nodes. The sequences of the *H. elani* clade were used to root the tree. The scale bars indicate the expected number of substitutions per site according to the model of sequence evolution applied. Lineage names in bold letters were found in accipitriform raptors. Species names indicate that the lineages were already linked to the respective morphospecies.
